# Image of Synthetic Biology and Nanotechnology: A Survey among University Students

**DOI:** 10.3389/fgene.2017.00122

**Published:** 2017-09-20

**Authors:** Christian Ineichen, Nikola Biller-Andorno, Anna Deplazes-Zemp

**Affiliations:** Institute of Biomedical Ethics and History of Medicine, University of Zurich Zürich, Switzerland

**Keywords:** ELSI, biotechnology, agricultural biotechnology, nanotechnology, synthetic biology, opinion, implicit, explicit

## Abstract

This study explores the image of synthetic biology and nanotechnology in comparison to agricultural biotechnology and communication technology by examining spontaneous associations with, and deliberate evaluations of, these technologies by university students. Data were collected through a self-completion online questionnaire by students from two universities in Switzerland. The survey aimed to capture implicit associations, explicit harm-benefit evaluations and views on regulation. The data suggest overall positive associations with emerging technologies. While positive associations were most pronounced for nanotechnology, agricultural biotechnology was attributed with the least favorable associations. In contrast to its positive result in the association task, respondents attributed a high harm potential for nanotechnology. Associations attributed to synthetic biology were demonstrated to be more positive than for agricultural biotechnology, however, not as favorable as for nanotechnology. Contrary to the evaluations of nanotechnology, the benefit-examples of synthetic biology were evaluated particularly positively. Accordingly, the investigated technologies enjoy different esteem, with synthetic biology and nanotechnology both showing a more “exciting” image. Even though, the image of nanotechnology was demonstrated to be more pronounced it was also more heterogeneous across tasks while agricultural biotechnology remains contested. For all technologies, the predominant spontaneous concerns pertain to risks rather than an immoral nature inherent to these technologies. Our data suggest that harm-benefit analyses reveal only one aspect of the attitude toward emerging technologies. Survey questions addressing spontaneous associations with these technologies are a valuable addition for our picture of the image of emerging technologies.

## Introduction

It is characteristic for emerging technologies in the 21st century that, from the very outset, their development and potential have been widely discussed by policy makers, academic scholars, the media, and the public. Policy strategies on these technologies have placed great importance on public opinion. Therefore, the Eurobarometer surveys, which are carried out regularly at the request of the European Commission, have included questions concerning controversial or emerging technologies (Gaskell et al., 2010). These data revealed, for instance, what views drove the controversies and public opposition against agricultural biotechnology (AGT). Meanwhile, studies on public opinions toward emerging technologies have been performed, indicating a generally positive perception of these technologies (Gaskell et al., 2010; Kronberger et al., 2012; Hacker et al., 2015). The latter suggest, at least for the European context, that nanotechnology (NT) has a comparatively positive image, and some researchers hypothesize that the image of synthetic biology (SB) might follow a similar pattern (Bainbridge, 2002; Schmidt et al., 2008; Torgersen, 2009; Gaskell et al., 2010; Kronberger et al., 2012; Shapira et al., 2015). Despite the first indications for positive reception of emerging technologies, there are concerns that these technologies might reignite past controversies and opposition as known from AGT (Greener, 2008; Marshall, 2009; Torgersen, 2009; Porcar et al., 2011; Torgersen and Hampel, 2012; Marris, 2015). Moreover, recent data indicates that the moral acceptability of SB could be contested (Akin et al., 2017). Unquestionably, more data are needed to understand how laypeople evaluate these technologies and what factors influence their evaluation.

Most surveys performed to date tend to be rather rudimentary by displaying a strong focus on explicit opinions, such as harm-benefit assessments. Although such standard harm-benefit evaluations are doubtlessly important, public perception and evaluation of emerging technologies are likely to involve other dimensions, such as emotions, values, taste, religious beliefs, or other types of moral norms. Simultaneously, there is mounting evidence of the influence of spontaneous, emotional processes in decision making as has been demonstrated by cognition theory (Smith and DeCoster, 2000). In the theoretical literature on emerging technologies, some of these aspects are discussed as concerns of “playing God” or “tampering with nature” (Dabrock, 2009; Van den Belt, 2009; Dragojlovic and Einsiedel, 2013; Link, 2013). Therefore, it comes as a surprise that, to our knowledge, empirical data on implicit aspects of the public perception of emerging technologies have never been collected.

This study aims at providing detailed quantitative data that allow us to better understand the current opinion among university students on SB and NT as two emerging technologies. Due to their young age, university students represent a population that frequently uses technological products (e.g., mass media). Another advantage of this cohort is that their comparatively high educational level increases the probability that they possess basic knowledge of recent technological developments. In addition, the different study subjects allow the identification of subgroups which may be expected to have different views regarding the importance of fostering innovation, associated risks and the like. University students are thus not only an accessible but also a particularly suitable source for initiating the investigation of the image of technologies. Further, we wanted to examine perceptions of technologies not only at an explicit level but also with respect to more implicit processes. For this purpose, we enquired after spontaneous reactions toward technologies. Based on such spontaneous associations we created – what we call – “technology-profiles.” We then combined these implicit opinions with explicit evaluations of deployment examples and opinions about how to regulate the technologies in question to derive the image of a technology. Even though, the term “image” refers to a multidimensional concept which is likely to include additional components, in the current work this three-component definition serves as an approximation to study the “image” of different technologies. It should be based on a wide set of evaluations and mirror a general perception that is based on deliberate (explicit) but also subconscious (implicit) processes.

To be able to integrate the opinions on emerging technologies into a wider picture of technology-perception, we compared responses concerning emerging technologies to two reference technologies [AGT and communication technology (CT)]. Finally, we analyzed effects of sociodemographic variables including academic background (students from natural sciences vs. students from humanities and social sciences – students from law, economics, medicine, and engineering have been excluded) and gender on the perception of emerging technologies.

## Materials and Methods

### Study Design and Rationale

This study investigates the image of the emerging (bio)-technologies, SB and NT in comparison to two reference technologies comprising CT and AGT. CT was selected as an example of a generally accepted modern technology, which became an essential component of most people’s private and professional lives. On the other hand, AGT was chosen as an example of a particularly controversial technology of the 20th century, which has been combated by systematic and institutionalized opposition (in German, the term “Grüne Gentechnik” has been used, which refers to the genetic modification of crops). We did not provide any definition of the investigated technologies because it is known that people construct preferences for topics even if they are not very familiar with them (Slovic, 1995, 2000). Furthermore, there is no clear and generally accepted definition for SB (Deplazes, 2009) or NT, and we wanted to avoid guiding participants by the framing of definitions. Because the opinion toward an attitude object (e.g., technology) is influenced by both spontaneous (implicit) and deliberate (explicit) processing levels (e.g., Conner et al., 2007; Karpen et al., 2012), we included both aspects in our survey. This furthermore aligns with recent insight from cognition theory positing how individuals process information at implicit and explicit levels (Smith and DeCoster, 2000). Finally, emerging technologies are rather unfamiliar to most people and hence not related to well-developed schemas and stored scripts (Fazio, 2007; Bekker et al., 2017). Therefore, it is worthwhile investigating automatic and spontaneous responses toward these technologies where the activation of pre-established attitudes is less likely.

**FIGURE 1:**
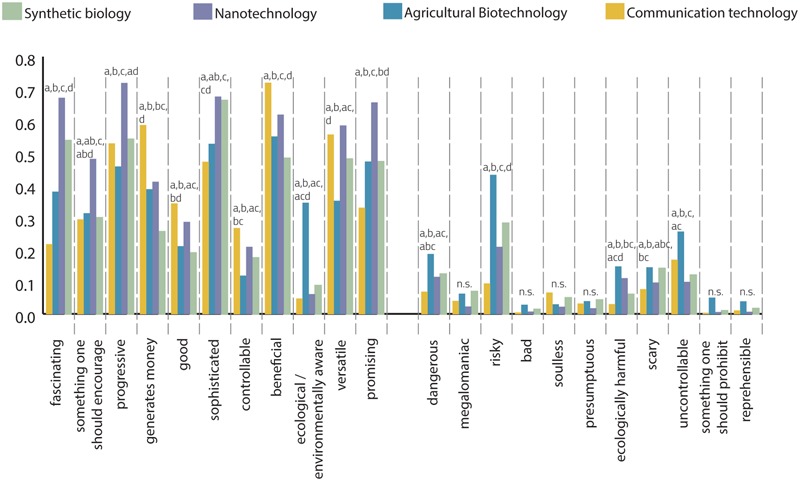
Bar chart of technology profiles: inter-technological differences on selection frequency of items based on a non-parametric Friedman test with pairwise *post hoc* comparison. To satisfy comparability, values have been normalized to the interval [0, 1] and represent the frequency of choice. Pairs of data points that do not share at least one letter are significantly different.

We asked participants to select those properties from a predefined set (see **Figure 1** and Supplementary Table S1) that they would spontaneously associate with the four technologies investigated in this study. We define the selected properties as ‘associations’ with the technology in question, and this contrasts with a well-considered balancing of pros and cons in the context of explicit evaluations of examples. It is known that people first form general perceptions toward a given technology and possibly infer from such general perceptions how harmful or beneficial they find a specific deployment (Frewer et al., 2004). Hence, we set the spontaneous association task at the beginning of the survey before participants were asked to perform other tasks. Because previous research highlighted the importance of assessing the public perception along concrete application examples (Siegrist et al., 2007; Siegrist, 2008), we also included an explicit harm-benefit assessment in our survey. Studies have shown that, in addition to the nature of applications, the type of organism involved influences the opinion toward a technology (Connor and Siegrist, 2013). To minimize biased responses based on moral objections against the application of technology to higher organisms, we used deployment examples in which the technology is directly applied only to bacteria and plants and not to animals. It has further been shown that technology assessment depends on the specific type of application being used to represent the technology (Frewer et al., 1997; Koivisto Hursti et al., 2002). In the harm-benefit task, we included applications for potential benefits and harm for humans as well as examples with such implications for the environment to increase inter-technological comparability of deployment examples.

To ensure that our deployment examples satisfy common quality characteristics, they were reviewed by experts regarding representativeness and relation to reality. Other studies indicated that harm-benefit perceptions drive beliefs about the acceptability of biotechnologies and thus requests for stronger or weaker regulation (Verdurme et al., 2003; Frewer et al., 2004). Finally, to assess this aspect, we asked participants what type and degree of regulation they consider suitable regarding the investigated technologies.

### Methods

The present study was performed with students enrolled in either the University of Zurich or the Swiss Federal Institute of Technology (ETHZ) in Switzerland. The survey invitation with the link to a self-completion questionnaire in German was sent out on April 14th, 2015, to all bachelor, master, and Ph.D. students through the university administration. The anonymous survey was developed with the help of Qualtrics,^1^ an online-survey software. An internal group reviewed and pre-tested the survey. The final questionnaire included three parts. First, we assessed demographic variables including study subject, gender, and year of birth. Besides, a simple test to assess participants’ basic knowledge of the underlying technologies was employed. In this knowledge task, we asked participants to assign eight sentences describing common practices and aims to each of the technologies. Whilst participants did only know that they had to allocate multiple sentences per technology, the correct type and number was unknown. In the mean, students made 6.7 out of 8 (or 83%) correct responses, indicating that the sample is informed and aware of the underlying technologies, consistent with our hypothesis. In the association task, we presented participants with a list of characteristic items and asked them to spontaneously select as many items (associations) as they thought to be characteristic for each of the different technologies (for associative structures and their influence on guiding behavior see Friese et al., 2008). We presented commonly used and easily comprehensible words as items (for translation into English, words were translated as precisely as possible while risking a varying degree of usability in the English language, see Supplementary Table S1). They were asked to perform this task as quickly as possible (as in the study by Friese et al., 2006; the mean per-item screening and potential selection time of our respondents was 1.7 s).

In the case of the implicit drag-and-drop task, the order of both the items and technologies was randomized across participants. The items were chosen based on discussions reported in the academic literature and media. The list of items has been completed until none of the authors was able to add any additional items to the list. For the explicit task, statements circumscribing deployment examples were pre-tested by experts working in the field of SB and NT. After the quality checking of 28 statements, 12 examples that satisfied the quality criteria (representativeness and relation to reality) were selected to represent the three technologies SB, NT, and AGT. Statements were presented one after the other in a 3x2x2 design (each technology × 2 conditions: effects on the environment and humans relating to potential harm and benefits). We asked participants to evaluate the degree of harm and benefit (for negative and positive framing, see Siegrist, 2010) for each statement using a five-point Likert scale ranging from “negligible” to “major.” For the analysis of this task, CT as a reference was excluded based on the assumption that harm is of a different nature (privacy and cybersecurity concerns rather than health or environmental damage) than those raised by the other technologies. In the last task dealing with the regulation of the four technologies, we asked students to assess the degree of regulation using a four-point Likert scale ranging from “general prohibition” to “no regulation.”

Informed consent was obtained from participants prior to performing the survey and respondents could quit the survey anytime. Among those participants who completed most of the survey (at least demographics and one task), eight book vouchers have been drawn. All studies in this research project were conducted in accordance with the legal situation in Switzerland stating that the study does not need mandatory authorization by the ethics committee.

We conducted descriptive statistical analysis using SPSS Statistics 24.0, complemented by inferential statistics including correlational analysis, non-parametric Mann–Whitney *U*-test, non-parametric Friedman test and ordinal regression with multinomial logistic regression, where necessary. Significance was accepted at a *p* < 0.05 level.

## Results

### Descriptive Analysis of Sample

In this study, we analyzed the responses of 1,474 students (response rate: 9.5%, 46.1% females), most of them studying natural sciences (53.7%) and engineering (26%) (**Table 1**). The over-representation of male students is mainly explained by the fact that, among the participants, more natural scientists and engineers answered the survey, where the number of male students still prevails. Moreover, students from the ETHZ, which mainly offers natural science and technology degrees, have responded more vigorously to the survey, favoring the mentioned gender distribution (for the ETHZ, participation rate is generally higher because special approval for distributing surveys is needed). The mean age of respondents was 23.8 years, and students have studied 6.3 semesters on average.

**Table 1:** Demographics of the respondents (*n* = 1474).

Mean age (years)		24
Gender (%)	Females	46.1
	Males	53.9
Study subject (%)	Humanities and social sciences	10.6
	Natural sciences	51.9
	Others	37.5

### Implicit Associations with the Examined Technologies

As described above, we explored participants’ associations with our four target technologies based on their implicit categorization of a set of presented items (for original items in German see Supplementary Table S1).

Generally, students associated more positive than negative items with the different technologies (**Figure 1**). Overall, NT received the most positive ratings (7,911 positive selections), whereas AGT received the fewest (6,058 positive selections). Consistent with our expectations, AGT received the most ratings by far on negative associations (2,072 negative selections), while CT received the fewest (861 negative selections; **Figure 1**).

In the following sections, we will use the phrase “technology profile” to describe how a technology is characterized by the selection of items on our list. A technology profile is thus determined by the selection frequency of positive and negative properties. We depict the technology profiles using a bar chart (**Figure 1**) and radar charts (**Figures 3, 5**). To compare technology profiles of different technologies, values that represent the frequency of choice have been normalized to the interval [0, 1]. For assessing inter-technological significant differences, we conducted the non-parametric Friedman test with pairwise *post hoc* comparisons (see **Figure 1**).

#### General Description of Technology Profiles

##### Technology profile of communication technology

In comparison to the other technologies, CT was statistically significantly more often associated with the items ‘beneficial’ and ‘generates money.’ On the other hand, participants significantly less often associate CT with the terms ‘fascinating’ (0.22), ‘promising’ (0.33), and ‘risky’ compared to SB, NT, and AGT. ‘Good’ and ‘controllable’ were significantly more often selected for CT compared to AGT and SB, while the CT ratings of these two items were not significantly different compared to NT.

##### Technology profile of agricultural biotechnology

Based on the strong opposition of environmental non-governmental organizations (NGOs) against AGT, it was somewhat surprising that the item ‘ecological/environmentally aware’ (from now on only referred to as ‘ecological’) was selected significantly more often for AGT than for the other technologies. With a three times higher association of this term for AGT, the difference from the other technologies was particularly pronounced. More expectedly, AGT showed the highest ratings regarding the items ‘risky’ and ‘uncontrollable’ to a statistically significant higher degree compared to the other technologies. The item ‘dangerous’ was also statistically more frequently associated with AGT compared to NT and CT but not compared to SB. This similarity between SB and AGT could also be observed for the item ‘controllable,’ which was significantly less often associated compared to NT and CT. From a total of 11 negative items, AGT received the highest ratings on eight of those compared to the other three technologies. Regarding ‘scary’ (0.14), AGT and SB shared a similarly high frequency of association, which was significantly different from that of NT and CT. Surprisingly, AGT showed a significantly increased association frequency for both items ‘ecological’ (0.34) and ‘ecologically harmful’ (0.15). Regarding the item ‘ecologically harmful,’ both AGT and NT showed a statistically significant increased selection frequency. Finally, we tested for similarity between AGT and SB and between AGT and NT. We found no statistically significant difference in item-attribution between SB and AGT in four of the 11 positive item-specific comparisons. In contrast, in only one of the 11 positive item-specific comparisons, there was no significant difference between NT and AGT. Regarding negative items, we found only five items for which there were any significant differences between the four technologies.

##### Technology profile of nanotechnology

In comparison to the other three technologies, NT is characterized by a significantly higher degree of association for the items ‘fascinating,’ ‘something one should encourage’ (0.48), ‘progressive,’ and ‘promising.’ Furthermore, NT yielded an association frequency that was significantly higher compared to two of the other three technologies for the items ‘sophisticated’ and ‘versatile’ (0.58). While there was no significant difference in the case of ‘sophisticated’ and SB, the same holds true for ‘versatile’ and CT. In general, from a total of 11 positive items, NT received the highest values compared to the other three technologies on six of them (and on 10 when excluding CT). Regarding the negative items, NT is attributed with the lowest or second lowest selection frequency in the inter-technological comparisons for all 11 negative items. However, ‘risky’ was the only item for which the difference reached statistical significance (compared to SB and AGT).

##### Technology profile of synthetic biology

Regarding the positive items, SB obtained second most frequent significant associations in the cases of ‘fascinating’ and ‘sophisticated’ and the lowest number of associations in the cases of ‘generates money’ and ‘beneficial.’ For positive associations, SB often played a middle role, not demonstrating the highest association frequencies in any inter-technological comparison. Regarding negative item selection, SB demonstrated the second-highest ratings after AGT for eight of the 11 inter-technological comparisons. However, in only two cases (‘risky’ and ‘scary’), the difference from NT and CT reached statistical significance.

Six of the 11 negative items have not been mentioned in the characterization of any of the four technology profiles because none of the inter-technological comparisons revealed any significant difference. Those terms include ‘megalomaniac,’ ‘bad,’ ‘soulless,’ ‘presumptuous,’ ‘something one should prohibit,’ and ‘reprehensible.’ In contrast, all the positive items appeared in one or the other description of technology profiles because, for all of them, there were characteristic differences in how they were associated with the different technologies. This is not to say that our description covered *all* significant deviations.

To evaluate the previously outlined descriptions with the required proportionality, please consult the bar chart (**Figure 1**).

#### Qualitative Differences

The description of technology profiles so far has focused on quantitative differences, meaning that we described differences in absolute selection frequencies of individual items between two or more technologies. Apart from these quantitative differences, we were also interested in comparing qualitative differences of associations for one technology relative to another. We computed an overarching measure of qualitative discriminability between the different technology profiles. While quantitative differences covered inter-technological comparisons of single items, qualitative differences tell us something about the overarching relations between the technologies. Thus, they are a measure of how similarly technologies were characterized, or in contrast, how qualitatively different the technology profiles are.

To exemplify, assume the following (simplified) thought example: while the item ‘fascinating’ was associated with AGT at a normalized frequency of 0.2 and with NT at 0.3, the item ‘generates money’ was associated with a frequency of 0.4 in the case of AGT and of 0.6 in the case of NT (see **Table 2**). In this scenario, there are no qualitative differences of the simplified two-item technology profiles because there is a common multiple (factor 2) across technologies and between items. Hence, qualitative differences can occur in the case of distinctive variations of common multiples (relation between different items). However, there would be quantitative differences in this example, when comparing the item ‘generates money’ between AGT and NT. Let us next assume the following frequencies for the same items when comparing AGT with SB: a shared, normalized frequency of 0.2 in the case of ‘fascinating’ relative to a frequency of 0.4 in the case of AGT and 0.9 in the case of SB for the item ‘generates money.’ Because there is no common multiple when comparing the simplified two-item technology profile of AGT and SB, in contrast to the previous comparison, there are now qualitative differences in addition to quantitative differences.

**Table 2:** Thought example to illustrate qualitative differences between technology profiles.

Technology	‘Fascinating’	‘Generates money’
AGT	0.2	0.4
NT	0.3	0.6
*No qualitative difference.*		
**Technology**	**‘Fascinating’**	**‘Generates money’**

AGT	0.2	0.4
SB	0.2	0.9
*Qualitative difference.*		

To evaluate qualitative differences in technology profiles between two technologies, we compared the selection frequency for each of the 11 items with all the other items, resulting in 55 comparisons. This strain of research is explorative in nature and represents an attempt to investigate such qualitative differences. For the quantification of qualitative differences, we computed differences of the selection frequencies of all items with each other to display selection variability for each item combination resulting in item matrices (see Supplementary Table S2). Since we were interested in finding variations of common multiples (relation between items), Pearson’s product-moment correlations, as a measure of shared common multiples, was performed. These results are visually represented in regular scatterplots. Due to the low selection frequencies of some of the negative items, the qualitative analysis was restricted to positive items.

##### Qualitative differences between technologies

**FIGURE 2:**
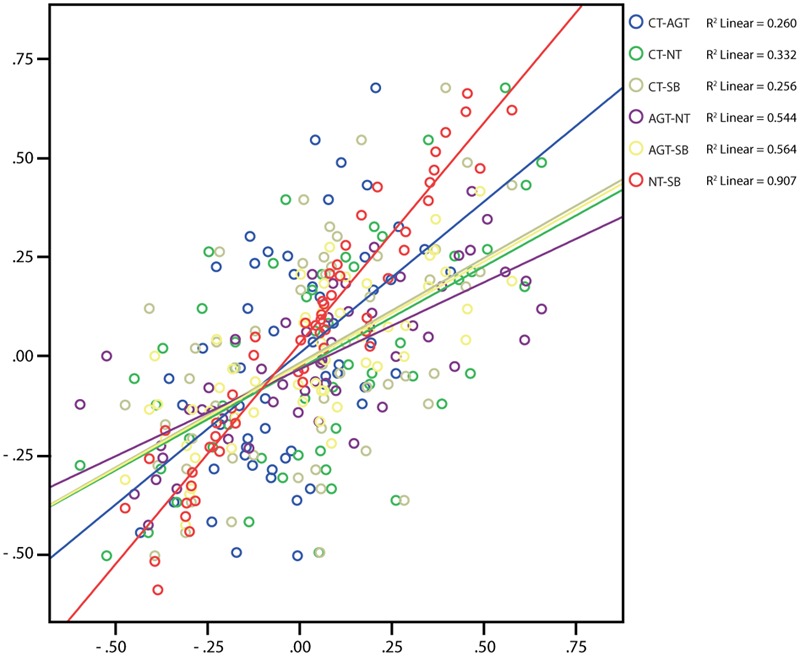
Scatterplot depicting Pearson’s Product-Moment Correlation for the differences of every association pair (each dot, *n* = 55 per comparison) from the sample of *n* = 1,473 students comparing association differences between technologies. The regression line as well as R is included in the graph.

Pearson’s product-moment correlations demonstrate only moderate positive correlations for technology combinations involving CT (CT-AGT: 0.510, CT-NT: 0.576, CT-SB: 0.506; all *p* < 0.0005) and strong positive correlations for the other comparisons (AGT-NT: 0.737, AGT-SB: 0.751, NT-SB: 0.952; all *p* < 0.0005) (for the interpretation of correlations see Evans, 1996). The first result indicated some degree of qualitative differences in the selection of items between the technologies because all correlations including CT show a significantly lower degree of correlation (**Figure 2**). Apart from degree to which technologies correlate, we were interested whether they share variance (covary). When inspecting the scatter plot, the regression models account for between 26 and 91% of variances. By far the lowest values are again attributed to comparisons with CT (i.e., when including CT, the model is least improved in explaining the total variance of the data).

#### Effects of Academic Background on Technology Profiles

**FIGURE 3:**
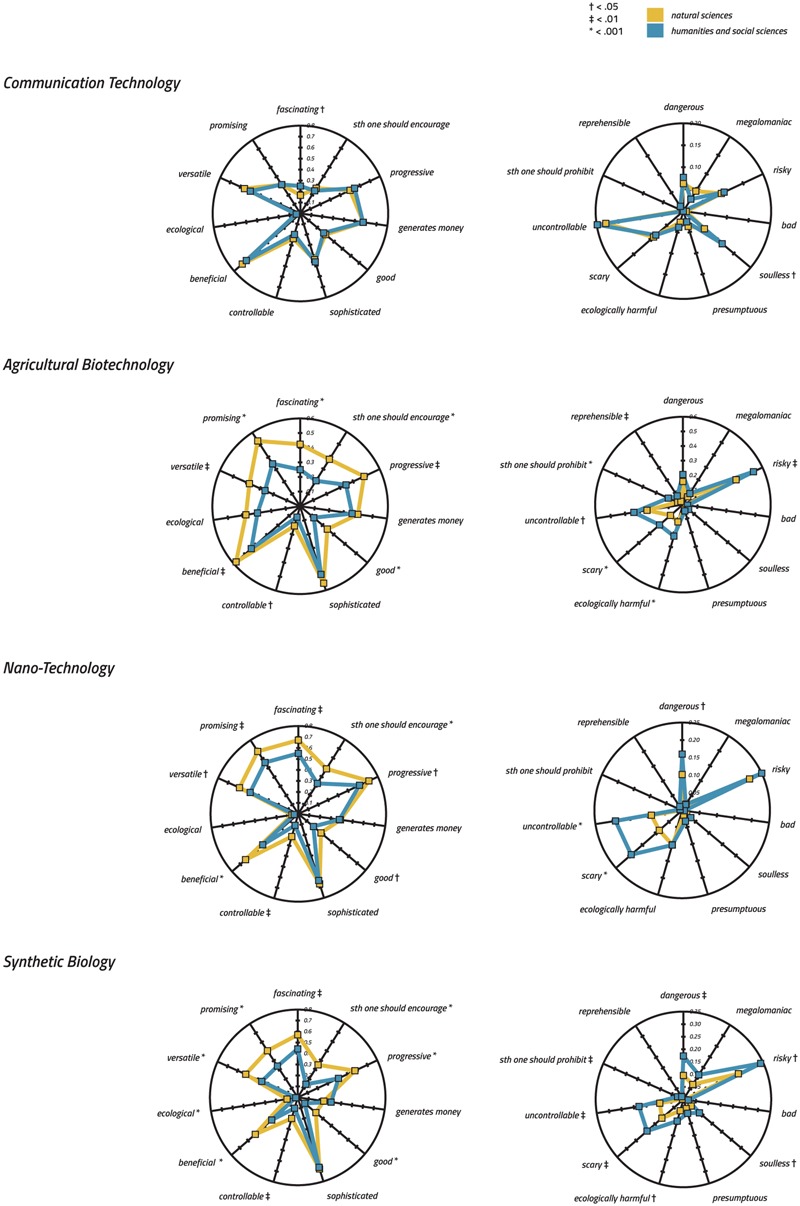
Radar charts showing quantitative effects of academic background on the technology profiles assigned to the four technologies (please note the varying Y-Axis). To satisfy comparability, values have been normalized to the interval [0, 1] and represent the frequency of choice. A non-parametric Mann–Whitney *U*-test has been used to demonstrate quantitative effects of academic background.

Since natural scientists are expected to be more interested in the science of the investigated technologies and to be confronted with more related background information than students in humanities and social sciences, the dominance of the former group of students may have distorted our results. We were therefore interested in how implicit associations relate to the academic background of respondents. We expected to find differences in technology profiles for the four technologies between the two subgroups because of different affinities and reservations in these student groups toward the technologies in question. More precisely, we assumed students from natural sciences to be more positive regarding the investigated technologies because, in most cases, they decided to study a subject that is directly associated with technological development. We thus compared the technology profiles assigned to the four technologies of students from humanities and social sciences with those of students from natural sciences. As illustrated by the radar charts (**Figure 3**), technology profiles of positive items for CT were almost identical for both student groups. In contrast, for SB, NT, and AGT, students from natural sciences indeed associated more positive items with the investigated technologies than students from humanities and social sciences. There is one interesting exception to this tendency, namely, the association of ‘fascinating’ in case of CT. Correspondingly, students from natural sciences associated less negative items with the investigated technologies than students from humanities and social sciences. However, for the sake of completeness, we want to add that, for ‘megalomaniac’ and NT and for ‘generates money’ and ‘reprehensible’ and SB, where selection frequency was very low, this tendency could not be confirmed.

For the quantitative analysis, we conducted a non-parametric Mann–Whitney *U*-test and found significant differences between groups for all investigated technologies (please consult **Figure 3** for specific effects of the group on the selection frequency of items). Interestingly, SB revealed the highest number of significant differences (i.e., disagreement) (*n* = 16 items), followed by AGT (*n* = 14), NT (*n* = 11), and CT (*n* = 2).

##### Qualitative differences between academic backgrounds

**FIGURE 4:**
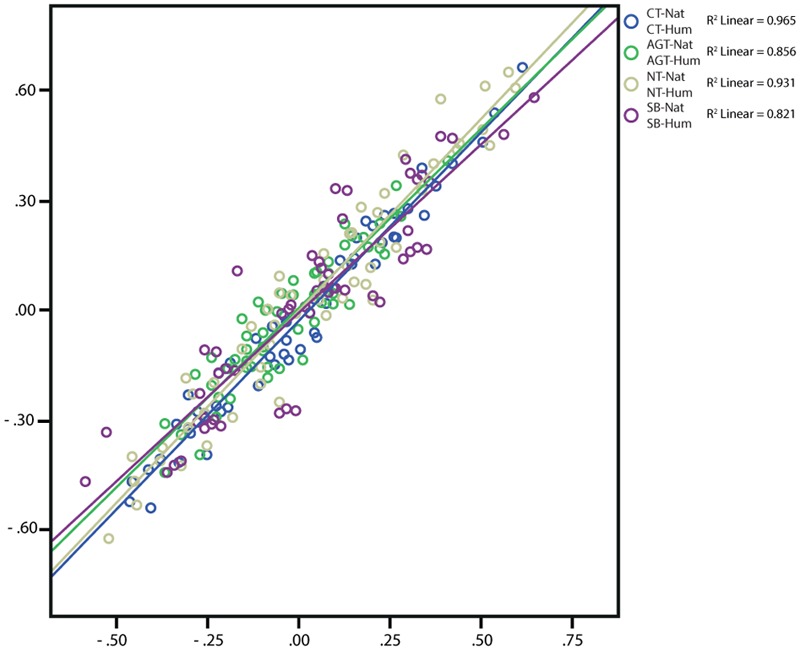
Scatterplot depicting Pearson’s Product-Moment Correlation for the differences of every association pair (each dot, *n* = 55 per comparison) from the sample of *n* = 1,473 students comparing association differences between academic backgrounds. The regression line as well as R is included in the graph.

Pearson’s product-moment correlations demonstrate very strong (Evans, 1996) positive correlations between students from both fields {CT-NatHum [natural sciences (Nat) humanities and social sciences (Hum)]: 0.983, AGT-NatHum: 0.925, NT-NatHum: 0.965, SB-NatHum: 0.906; all *p* < 0.0005} (**Figure 4**). These results indicate that qualitative differences in the selection of items between the two subgroups are rare for all technologies. When inspecting the scatter plot, the regression models account for between 82.1 and 96.5% of variances. Hence, all models reduce error substantially and explain the large majority of variance (lowest: SB). Contrary to the results collected from the comparison between the different technologies (see above), academic background seems to influence qualitative technology profiles to only a low degree. Accordingly, the correlations between the two backgrounds with respect to one technology are stronger than the correlation between the different technologies as depicted in **Figure 2**, indicating more qualitative similarity between students from different academic backgrounds than between the investigated technologies.

#### Effects of Gender on Technology Profiles

**FIGURE 5:**
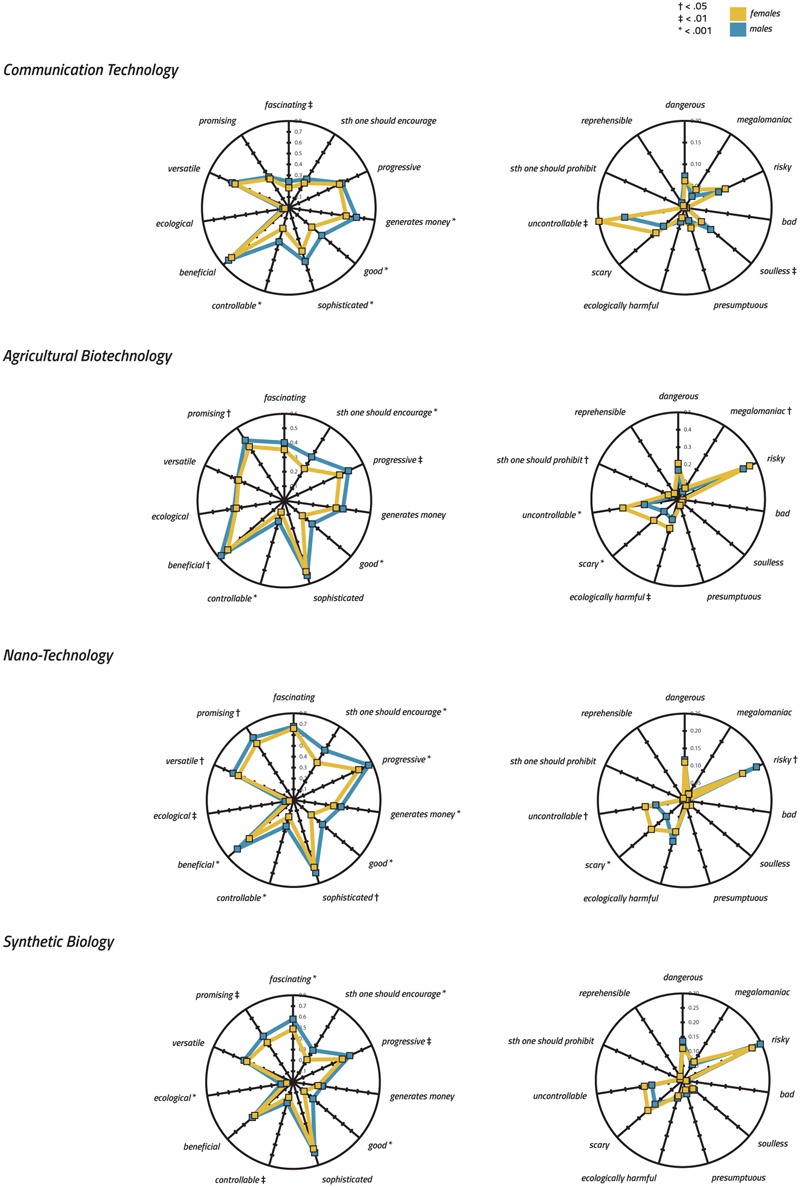
Radar charts showing quantitative effects of gender on the technology profiles assigned to the four technologies (please note the varying Y-Axis). To satisfy comparability, values have been normalized to the interval [0, 1] and represent the frequency of choice. A non-parametric Mann–Whitney *U*-test has been used to demonstrate quantitative effects of gender.

Because it was previously reported that gender influenced technology evaluation (Siegrist, 2003, see Davidson and Freudenburg, 1996, for a review), we examined whether the implicit task also reveals such an effect. Indeed, we found that female students clearly selected less positive items and tended to select more negative items than male students (see the radar chart for specific results; **Figure 5**). In contrast to the comparison of academic background, a substantial number of differences can also be observed for CT when comparing males with females. Exceptions include the ‘risk’-attribution in the case of NT and the selection of the item ‘soulless’ in the case of CT, in which selection frequencies between male and female students were reversed. The effect of gender was more pronounced for positive than for negative items (again partly explained by the fact that absolute numbers for negative associations were lower). As an exception, for AGT, five of the 11 comparisons in which significant differences emerged were devoted to negative items. Being the technology with the highest number of negative associations, AGT was also the only technology for which the gender effect for positive and negative items was almost the same.

##### Qualitative differences between gender

**FIGURE 6:**
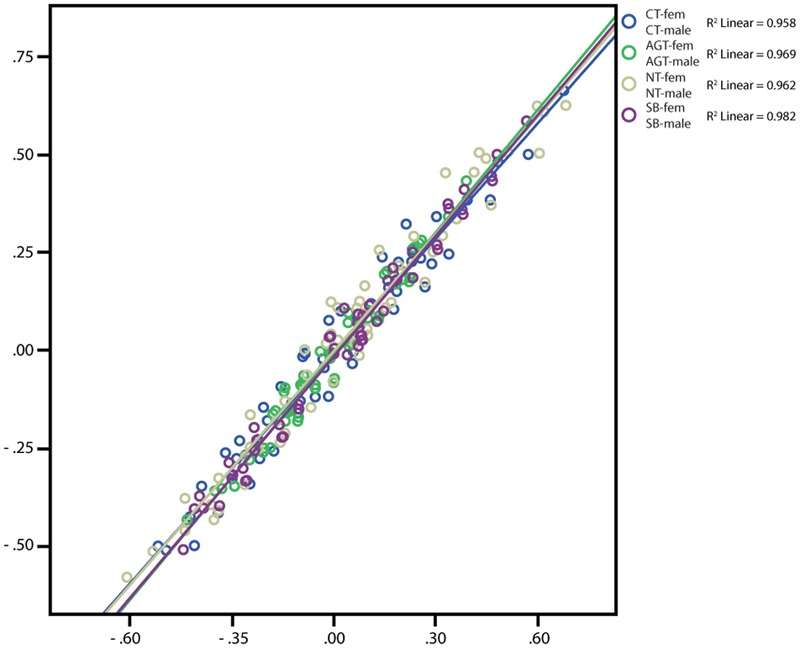
Scatterplot depicting Pearson’s Product-Moment Correlation for the differences of every association pair (each dot, *n* = 55 per comparison) from the sample of *n* = 1,473 students comparing association differences between gender. The regression line as well as R is included in the graph.

Pearson’s product-moment correlations demonstrate very strong (Evans, 1996) positive correlations between females and males (CT-FM: 0.979, AGT-FM: 0.984, NT-FM: 0.981, SB-FM: 0.991; all *p* < 0.0005) (**Figure 6**). These results indicate that association patterns between male and female students are very similar, and qualitative differences are neglectable. In the scatter plot, the regression models account for between 95.8 and 98.2% of variances. Hence, all models explain the majority of variance (lowest variance between males and females: CT). Similar to the comparison of academic background, there was a neglectable effect of gender in qualitative differences of the technology profiles. In sum, pronounced qualitative differences emerge neither in case of a potential effect of academic background nor gender. Rather, the inter-technological comparison seems to reveal most detectable differences.

### Students Explicit Harm-Benefit Evaluations

**FIGURE 7:**
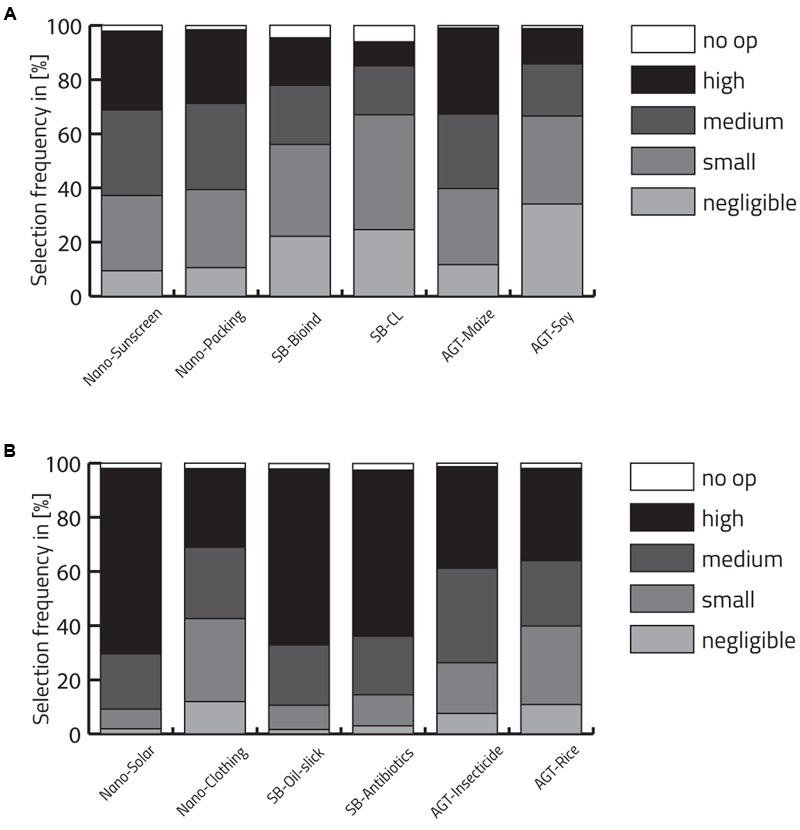
Aggregated evaluations of six statements on harm and benefit potential of AGT, SB, and NT along a five-point Likert scale ranging from negligible to high including the option to select ‘no opinion.’ **(A)** List of statements, harm potential: NT: the harm potential of sunscreen nanoparticles escaping into the environment (Nano-Sunscreen)/the harm potential of nanoparticles in food packaging for humans (Nano-Packing). SB: the harm potential of synthetic biologically modified bacteria in form of bio-indicators for heavy metals released into the environment (SB-Bioind)/the harm potential of synthetic biology in the context of closed loop systems for the recognition of cancer cells in humans (SB-CL). AGT: the harm potential of biotechnologically modified maize with intrinsic ability to defend against vermin (AGT-Maize)/the harm potential of biotechnologically modified food products (e.g., soy products) for humans (AGT-Soy). **(B)** List of statements, benefit potential: NT: the benefit potential of nano-technologically produced solar cells for the environment (Nano-Solar)/the benefit potential of nano-technologically fabricated sports-clothing for humans (Nano-Clothing). SB: the benefit potential of synthetically modified oil-slick-degrading bacteria for the environment (SB-Oil-slick)/the benefit potential of synthetic biologically fabricated antibiotics fur humans (SB-Antibiotics). AGT: the benefit potential of biotechnologically modified maize to reduce herbicides/insecticides and hence minimise environmental pollution (AGT-Insecticide)/the benefit potential of biotechnologically modified rice with increased levels of pro-vitamin A for humans (AGT-Rice).

To explore the explicit opinion of study participants toward applications of AGT, NT, and SB, we analyzed their deliberate harm-benefit assessment of characteristic applications for humans and for the environment. For this purpose, they were asked to evaluate characteristic statements that have been pre-tested (see Methods). We are aware of the fact that generalizations about the underlying technologies and thus inter-technological comparisons are problematic if statements do not satisfy quality characteristics of classical prototypes. Because of the potential influence of the choice of examples used as stimulus material, we specify the technology in terms of domain of application rather than making general statements about the harm and benefit assessment of the technology as a whole (e.g., not NT in general but the benefit of NT for environment related to solar cells; for statements see legend of **Figure 7**, for original statements in German see Supplementary Table S3).

Respondents generally estimated a greater benefit than harm potential for the deployment examples of the different technologies (**Figure 7**). The benefit estimation was particularly high for the environment in case of solar cells produced by NT and for both benefit statements of SB (environmental benefit of oil-slick-degrading bacteria generated by SB, and medical benefit for humans of new antibiotics produced by SB). The highest harm potential was attributed to both NT examples (damage potential of sunscreen nanoparticles escaping into the environment and the harm potential of NT in food packaging for humans) and to the AGT example concerning environmental effects (maize that was genetically modified to express a toxin against vermin).

#### Effects of academic background and gender on explicit statement task

**FIGURE 8:**
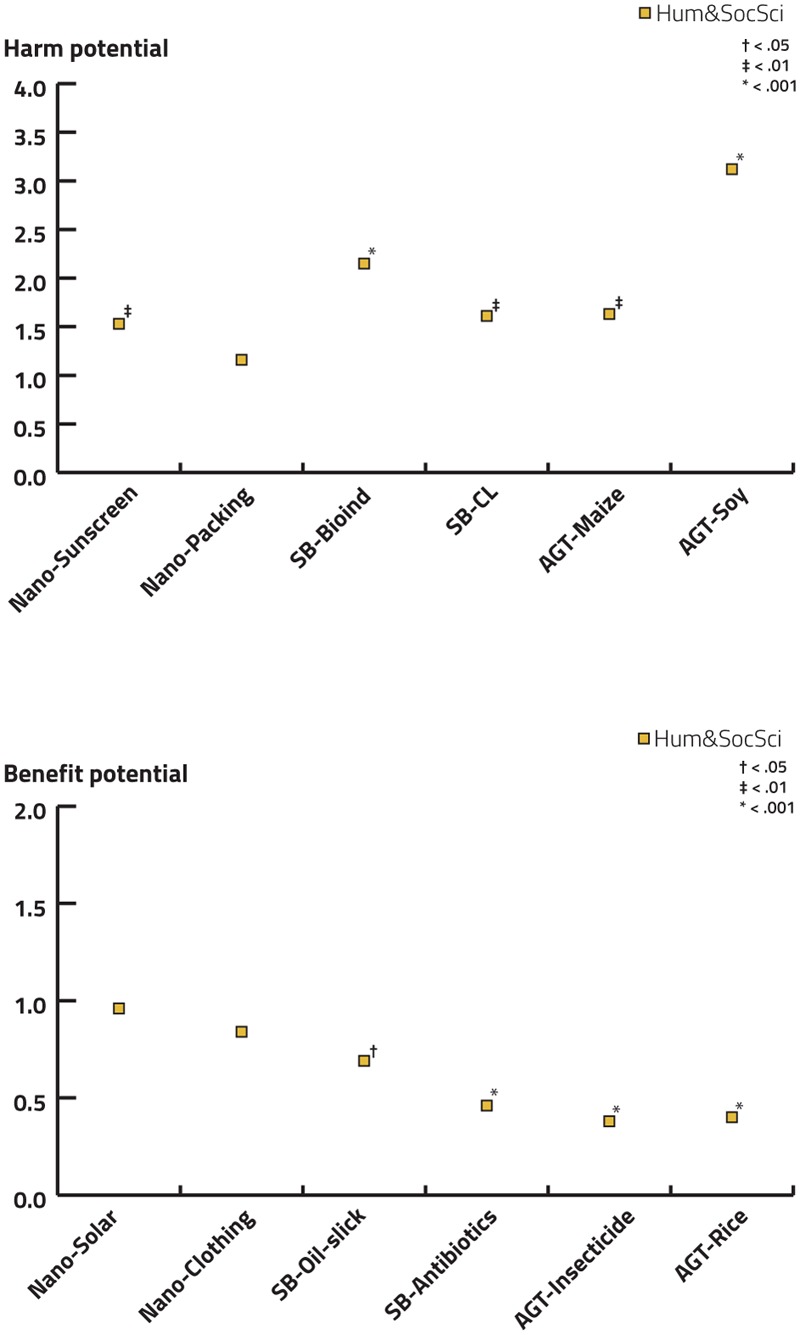
Effects of academic background on the estimation of harm and benefits in the deployment examples for NT, SB, and AGT using ordinal regression models. If models did not satisfy the test of parallel lines, a multinomial logistic regression was performed. As a reference category, natural sciences were selected. Significant results are indicated.

To investigate whether the academic background (humanities and social sciences vs. natural sciences) influences the evaluation of benefits and harm potentially associated with the different technologies, we performed ordinal regression (**Figure 8** and Supplementary Tables S4, S5 for details). If models did not satisfy the test of parallel lines, multinomial logistic regression was conducted. As reference category, natural sciences were selected.

Compared to students from natural sciences, students from humanities and social sciences generally estimated the harm potential of the investigated technologies to be higher and the benefit potential to be lower. The odds of students from humanities and social sciences considering the harm potential of the example of sunscreen nanoparticles escaping into the environment to be high, was 1.53 times that of students from natural science (*p* < 0.01). In contrast, and as an exception to the general tendency, the evaluation of the two student groups did not significantly differ with respect to the example describing a harm potential of NT in food packaging for humans. For both SB examples, the tendency of a more critical perception of students in social sciences and humanities was clearly confirmed. The tendency was particularly pronounced in the case of the example for a potential harm to the environment (environmental release of microorganisms produced by SB as bio-indicators of heavy metals; odds: 2.15; *p* < 0.001). Students from humanities and social sciences were also more alarmed when reflecting the harm potential of SB in the context of a closed loop system for the recognition of cancer cells in humans (odds: 1.61, *p* < 0.01). The greatest difference between the two groups could be observed for the example discussing the harm potential for humans in the context of AGT [genetically modified soy products in human food products (odds: 3.12; *p* < 0.001)]. In contrast to SB, this difference was more pronounced regarding the harm potential for humans than the harm potential for the environment enquired with the example of genetically modified maize to express a toxin against vermin (odds: 1.63; *p* < 0.01). This is not to say that students in humanities and social sciences perceived the potential that this example could be harmful as less severe but rather that the difference was not as pronounced because already students in natural sciences have associated a high harm potential with this example.

In terms of participants’ benefit evaluation, students from humanities and social sciences were significantly less likely to evaluate the examples of SB (synthetic biologically engineered oil-slick-degrading bacteria and synthetic biologically derived antibiotics) as beneficial compared to students from natural sciences (odds: 0.69; *p* < 0.05, 0.46; *p* < 0.001, respectively). The same holds true for both examples of AGT (modified crops to reduce herbicides/insecticides and modified rice with increased levels of pro-vitamin A) (odds: 0.40; *p* < 0.001, 0.38; *p* < 0.001). There were no significant differences with respect to academic background and explicit benefit assessment of NT.

**FIGURE 9:**
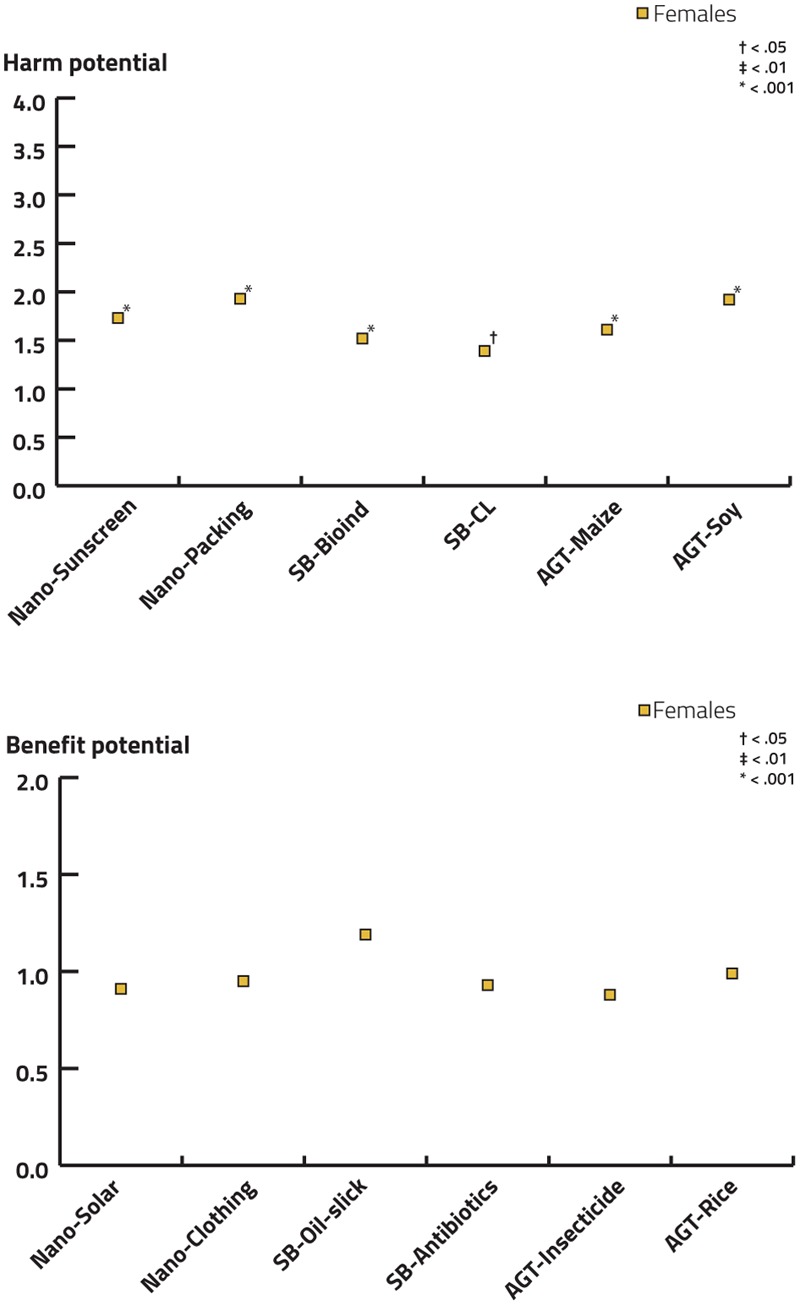
Effects of gender on the estimation of harm and benefits in the deployment examples for NT, SB, and AGT using ordinal regression models. If models did not satisfy the test of parallel lines, a multinomial logistic regression was performed. As a reference category, males were selected. Significant results are indicated.

When investigating effects of gender, females attributed a higher harm potential to all technological deployments of any technology than males while no significant differences emerged regarding the benefit attribution (see **Figure 9** and Supplementary Tables S4, S5 for details).

Hence, by performing ordinal regression to test academic background and gender as determinants of explicit technology assessment we found significant effects of both variables on the harm-benefit evaluation of statements. Although only small effect sizes were observed (in the mean Nagelkerke *R*^2^ was 4%), it is interesting to emphasize that gender and academic background significantly contribute to the harm-benefit evaluation. It is not surprising that these two variables only partially predict the harm-benefit evaluation since there are many other predictors that are likely to exert influence on the outcome variable, for example, a person’s self-assessed status (Connor and Siegrist, 2013), religious belief, deference to scientific authority, or trust in scientists (Akin et al., 2017) among others.

### Participants’ View on Regulation

**FIGURE 10:**
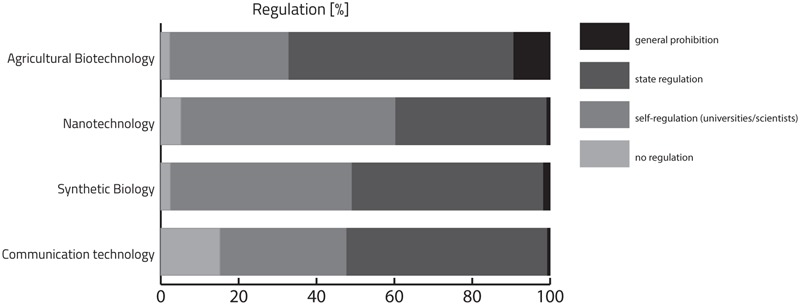
Descriptive analysis of participant’s view on regulation (%) of AGT, SB, NT, and CT using a four-point Likert scale ranging from “no regulation” to “general prohibition.”

At the end of the questionnaire, we assessed participants’ views on regulation of the enquired technologies (**Figure 10**). As may be expected from the public opposition against AGT, university students also wanted this technology to be most strictly regulated, with nearly 10% opting for general prohibition and another 58% opting for state regulation. While NT received the most liberal assessments, with only 1% choosing general prohibition and 55% opting for self-regulation by universities or scientists, SB was divided by approximately 50% of participants opting for general prohibition/state regulation or self-regulation/no regulation, respectively. A similar pattern could be observed for the selection of regulation options in the case of CT. However, while in the case of SB, the large majority of those who were against state regulation still requested self-regulation, in CT, nearly 1/6 of all respondents opted for no regulation.

To investigate the influence of background on degree of regulation, we again conducted ordinal regression (Supplementary Table S6 and Figure S1). The odds that students from humanities and social sciences consider that AGT should be prohibited was significantly increased by 2.2 times [95% confidence interval (CI), 0.400 to 1.139] that of natural scientists. In line with this, the odds that students from humanities and social sciences consider that NT and SB should be prohibited was 2.4 times (95% CI, 0.529 to 1.258) and 2.1 (95% CI, 0.378 to 1.115) that of natural scientists. There was no statistically significant difference in the odds of students from humanities and social sciences in the case of considering prohibition for CT.

Finally, differences relating to gender revealed that males were significantly less strict in their opinion regarding how to regulate both AGT and CT compared to females. Interestingly, for SB and NT, there were no statistically significant differences (Supplementary Table S6 and Figure S2).

## Discussion

The evaluation of both tasks – the implicit associations (technology profiles) and the explicit harm and benefit assessment – indicate a predominantly positive opinion amongst university students toward the investigated technologies and thus reveal that the technologies have attained a generally positive image. This general tendency is not due to the higher number of students from natural sciences since, albeit less pronounced, this tendency can also be observed if only responses from females and students in social sciences and humanities are considered. Our results thereby confirm previous findings (Gaskell et al., 2010; Kronberger et al., 2012; Hacker et al., 2015) of a generally positive perception toward emerging technologies and are thus in line with previous suggestions speaking of a ‘departure of the crisis of confidence’ as a result of the regulatory failures in the 1990s (Gaskell et al., 2010). In the following sections, we discuss the specific findings of the implicit and explicit tasks in more detail.

### Spontaneous Associations with Technologies in the Implicit Task

Our data reveal that, in general, emerging technologies evoke, at least among the investigated sample positive notions of fascination, technological progress, sophisticated techniques, a high degree of usefulness apart from versatility and a promising nature. On the other hand, fewer negative items were selected and if so, they were characterized by a lower selection frequency compared to most positive items. Among the negative associations concerns about risks, harm and uncontrollability clearly predominated. The item ‘uncontrollable’ was even more frequently selected for CT than for SB and NT, although one might expect that the new technological possibilities anticipated for emerging technologies would raise more concerns of uncontrollable consequences. It is worthwhile considering the high attribution of uncontrollability for CT in the context of its omnipresence in daily life, which may very well relate to cybersecurity, for instance. In accordance with this everyday effect, CT has been assessed as beneficial, economically interesting, and non-risky. In addition, AGT fulfilled our expectations as a reference technology by being described as the most risky, uncontrollable, and potentially dangerous of the four technologies. The latter also mirrors its history of public concerns (Gaskell et al., 2010). Moreover, participants attributed a lower level of fascination, versatility, or promising prospects to AGT compared to other technologies. However, we also find indications for a potential image change of AGT, for instance, when participants associated AGT not only with ecological harm but also with ecological benefits to a significantly higher extent compared to the other technologies. Regarding NT, participants expressed their particularly high level of support (reflected in the item ‘something one should encourage’), high level of fascination, and a belief in the technology’s promising future advances. A general lack of negative associations substantiates the positive technology profile of NT. Interestingly, even though SB raises higher fascination than AGT or CT, participants question its usefulness, perceive it as economically least attractive and implicitly associate a higher risk-potential with it (second highest after AGT). Regarding qualitative differences, inter-technological correlations are lowest if CT is included, while the differences of item pairs correlate most strongly between the two emerging technologies NT and SB.

We next discuss quantitative and qualitative effects of academic background and gender on university students’ implicit associations with the four investigated technologies. In terms of quantitative effects, students from natural sciences were generally more positive than students from humanities and social sciences in their evaluation of NT, SB, and AGT, while female students were more skeptical than male students. The fact that we found consistent results regarding quantitative effects of group (e.g., for all three technologies, females were more skeptical) corroborates the robustness of the found effects. In contrast to these quantitative differences in technology profiles, qualitative differences of the groups are negligible; we find a substantially high degree of similarity, as evidenced by the high inter-group correlations. Hence, qualitative differences between the technologies are more pronounced than when analyzing effects of academic backgrounds and/or gender. This means that students of both academic backgrounds and genders attributed to a large degree the same association pattern to a technology (qualitative similarity) but that this tendency was quantitatively less pronounced in female students and students from humanities and social sciences. This observation may be relevant in policy making, if one thinks, for instance, of measures to promote or regulate these technologies. Based on our implicit results, we would expect that the different groups would spontaneously agree on what measures are appropriate but not on the urgency of implementing them. A comparison with a more dissimilar group (i.e., laypeople or senior adults) will be needed to verify this outcome.

### Harm-Benefit Assessment of the Technologies in the Explicit Task

In accordance with the results of the implicit task, the explicit task also revealed that participants tended to attribute higher benefit potentials than harm potentials to the selected deployment examples. This tendency is particularly pronounced for SB and least pronounced for NT. The harm potential for both deployment examples of NT were evaluated as comparably high, and the benefit potential for the example of nano sport cloths was evaluated as low (the latter can be explained by the apparent lack of comparability relative to the other examples used). This outcome is somewhat in tension with the particularly positive outcome of NT in the association task and could be interpreted as a change in perception when lowering the level of abstraction. However, because our deployment examples lacked the quality of strict prototypes, inter-technological comparisons (and strictly speaking also net comparisons between benefit and harm assessments) should be carried out with the utmost caution.

Consistent with the quantitative results of the implicit task, the evaluation of deployment examples also revealed the same effects of academic background and gender. Students from humanities and social sciences attributed a higher harm and a lower benefit potential to the different examples, while females attributed a higher harm potential to the investigated technologies only. Again, the former effect was least pronounced for NT.

To consider the evaluation of potential harms and benefits for both humans and the environment, we included both types of benefit and harm in each technology deployment example. We could only partly support previous findings reporting a higher acceptance and ascription of utility to medical compared to agricultural applications of biotechnology (Dietrich and Schibeci, 2003; Bhattachary et al., 2010). In addition, our data show a high benefit attribution for environmental examples. Notably, the highest benefit potential of all our examples was attributed to the case of environmental benefits of NT (nano-solar cells for the environment). This was also confirmed by other studies indicating that energy applications of NT are perceived positively (Siegrist, 2010). However, we also find support of high acceptance of medical products in the case of the SB example. Taken together, our data support the notion that, in addition to medical applications, ecological applications receive high benefit estimations. Moreover, even though people discriminate between naturally occurring and technological risks (Siegrist, 2003), different applications are assessed differently.

### Comparing the Results from the Implicit and Explicit Tasks

As discussed above, both tasks point to a generally positive opinion toward emerging technologies, which, as may be expected, is particularly pronounced among male students from natural sciences. To compare the implicit and explicit outcomes, we selected those associations from the implicit task that relate to direct harm (‘risky’ and ‘dangerous’) and benefit (‘beneficial’ and ‘generates money’) and compared their attribution to the evaluation of the explicit statements on harm or benefit potential. For the items ‘beneficial’ and ‘generates money’ in the implicit task, the significant inter-technological differences (SB, in both cases lowest values) are not mirrored in the data of the explicit task, which showed a predominantly positive assessment of benefits for the SB examples in particular. This result indicates assessment instability between more spontaneous and deliberate reactions. Hence, while implicit usefulness attribution was significantly higher in the case of AGT compared to SB, the pattern reversed when decreasing the level of abstraction and providing participants with concrete examples. Another difference between the two tasks could be observed in the case of negative consequences for NT. While NT was considered by far the least risky and least dangerous technology in the implicit task, the harm potential in both deployment examples was considered higher than that for both SB examples and even higher than the harm potential of AGT for humans. These results indicate that the implicit task may not only provide information on associations with the underlying technology that cannot be grasped by a harm-benefit analysis but also that implicit tasks may complement the responses on harm-benefit evaluations to explicit examples. There is evidence from social psychological research positing that a low affective-cognitive inconsistency and hence increased attitude strength is more likely to predict behavior (Karpen et al., 2012). Correspondingly, the observed assessment instability between implicit and explicit results in the case of usefulness ascription of SB or that NT was associated most positively but showed the highest harm attribution in the explicit task may be based on the emerging nature of the underlying technologies and that the consideration of additional information about the attitude object eliminates the effect of automatic associations (Gawronski and LeBel, 2008). Hence, outcomes might be less predictive than in the case of AGT, where results between the two tasks better match. Unfortunately, data on the predictive validity of implicit and explicit measures is scarce (Friese et al., 2008). Whether implicit measures can predict behavior when controlling for explicit attitudes revealed mixed results (e.g., Ayres et al., 2012; Cameron et al., 2012). In addition, these processes certainly depend on situational factors, such as the scarcity of cognitive resources and the amount of processing time, among others. Hence, in situations where cognitive resources are depleted, such as when people are tired, one would speculate that participants’ implicit thoughts would predominate, whereas the opposite would be true if time for consideration is available and people can rely on pre-established schemas of known objects. Since emerging technologies are not “known” and it is unlikely that people have well-developed cognitive scripts at their disposal, more thorough implicit investigations would be an important undertaking to investigate predictive opinions of individuals until information on these technologies is more widespread and evaluations get increasingly enriched by explicit considerations. In summary, based on this insight from social psychology, we would be tempted to put slightly more weight on the implicit outcome for NT and SB.

As one aim of this study was directed at examining and comparing the image of the two emerging technologies SB and NT, in the following sections, we will discuss the characteristics of students’ opinions toward SB and NT in more detail.

### Results Relating to Synthetic Biology Specifically

The positive association ‘sophisticated’ has been selected most frequently for SB (66.4%), followed by the items ‘progressive’ and ‘fascinating’ (54.3% and 53.9%, respectively). These items seem to be characteristic for emerging technologies, as they have been attributed to NT with similar (‘sophisticated’) or higher (‘fascinating’ and ‘progressive’) frequencies. A great deal of fascination for SB has also been observed in other studies (e.g., Bhattachary et al., 2010). In the age group that has answered the survey, this may be related (among other explanations) to the playful way scientists convey the creative possibilities of this technology (as exemplified by the student competition iGEM). Notably, it was not only natural scientists who were fascinated by SB, as this item represents the second most often selected item of students from humanities and social sciences. Likewise, this generalized fascination of SB includes both genders.

The items ‘beneficial,’ ‘generates money,’ and ‘promising’ were significantly less frequently selected for SB compared to NT, and in the case of the former two, even significantly less frequently for SB compared to AGT. As mentioned above, this contrasts with the results from the explicit deployment evaluation where respondents attributed a medium to high level of benefit potential to both deployment examples of SB (benefit “medium-high” for the environmental example at 89% and human health example at 85%). With respect to the scary and risky nature that was implicitly associated with SB, we found a contrasting tendency in the explicit task, where a particularly low level of harm potential was associated with both SB examples (environmental example “negligible-small” at 59% and human example at 71%).

A particularly interesting question concerning our technology comparison is whether the technology profile of SB is more similar to that of AGT or NT. Since SB, like AGT, represents a biological type of technology, it may be closely associated with AGT by laypeople. Accordingly, some experts discussed that SB might reignite past controversies and debates relating to gene technology (Greener, 2008; Marshall, 2009; Torgersen, 2009; Porcar et al., 2011; Torgersen and Hampel, 2012; Marris, 2015). Alternatively, the image of SB might be more similar to that of NT, which has retained a comparatively positive image in Europe (Schmidt et al., 2008; Torgersen, 2009; Kronberger et al., 2012; Shapira et al., 2015). To compare the similarity between SB and AGT and between SB and NT, we analyzed the results of the Friedman test to detect similarity in the implicit task (no statistical difference is indicative for similarity). We found the associations of SB to be more similar to NT than to AGT (SB-NT: 7 statistical indifferences; SB-AGT: 6). In addition, we used the same test to examine whether SB or NT was more similar to AGT. The results indicate that absolute numbers of associations with SB were more similar to AGT than those of NT to AGT due to a higher number of statistically indifferent results (6 indifferences for SB-AGT vs. 3 for NT-AGT). Unfortunately, there is no clear evidence from the investigation of qualitative differences that could demonstrate a stronger correlation between AGT and SB compared to NT (the former explaining only 2% more of the total variance).

### Results Relating to Nanotechnology Specifically

As in the case of SB, we now proceed to discuss in more detail what our results reveal about the image of NT. In the implicit task, NT stood out with the most positive results. Accordingly, the association frequency of NT was highest regarding positive item selection and lowest in the case of negative item selection compared to SB and AGT in the implicit task. Surprisingly, NT was associated with even more positive items than CT, our positive reference technology. The positive opinion toward NT is in line with other studies that used representative samples that stress the wide interest and positive assessment of NT (Bainbridge, 2002; Gaskell et al., 2010). In contrast to this positive evaluation in the implicit task, participants were more critical in the explicit task. The harm potential of both presented deployment examples was evaluated as greater than in the cases of AGT and SB. The harm assessment of the explicit environment example for NT seems to relate to participants’ implicit association with ‘ecologically harmful’ (which was the third most selected negative item for NT). In contrast, participants also expressed a high benefit assessment of NT in environmental applications, such as nano-fabricated solar cells (91% assessed this example to be of moderate to high benefit), even though ‘ecological’ was rather rarely selected by participants in the implicit task. In contrast to our data for SB, we did not observe any significant effect of academic background for the explicit benefit evaluation of NT deployment examples and for the harm potential of NT in food packaging. This result may be related to reports in the media and scientific papers, where examples of potential harm caused by nanoparticles that enter the human body had also been discussed critically (Maynard et al., 2006; Nel et al., 2006; Helmus, 2007). The fact that no significant difference between academic backgrounds has been found in these examples together with the fact that correlational analysis testing for qualitative differences of the implicit task showed stronger correlations between the two groups of academic backgrounds in the case of NT relative to SB (and quasi-similar correlations when analyzing effects of gender) might indicate that for NT, responses between groups were more uniform as compared to SB. This uniformity is yet challenged by a more heterogeneous assessment of NT across tasks (implicit, explicit, regulation). In addition, the qualitative inter-technological analysis revealed that the SB and NT technology profiles share a higher degree of similarity as compared to the two reference technologies.

Regarding regulation of NT, about 40% of respondents opted for state regulation, while an even larger group supported the idea of self-regulation by universities or scientists (55%). These findings are in line with previous reports, stating that 52% of European citizens believe that the technology should be governed by the principle of scientific delegation (scientific experts) (Gaskell et al., 2010). The requirements for regulation were least stringent for NT compared to the other three technologies, which corresponds to NT’s positive technology profile. As expected, most rigorous regulation was requested for AGT where almost 10% of the respondents opted for general prohibition (9.5%) and almost 60% for state regulation (57.8%).

### Limitations

Since our research sample consists of university students (mostly engineers and natural scientists) generally interested in technological developments, there may be a bias toward more positive perceptions in our study. However, we addressed this limitation by analyzing effects of academic background and could demonstrate that the potential bias does not affect our main statements. For general conclusions about the public view on emerging technologies the fact that our sample consists of young and well-educated people means that our results cannot be directly extrapolated to the public because previous reports documented that students and those who hold a scientific worldview have a greater inclination to declare positive perceptions on biotechnological applications and are less worried about risks (Eurobarometer, 2005). Because the answers were collected mainly from Swiss citizens, we are unable to make statements about effects potentially driven by cultural determinants. Some of our general results have been confirmed by other studies with more representative samples, but to address this limitation, the implicit task should be repeated under laboratory conditions and with another age group. In general, to make more conclusive statements about the comparison of implicit and explicit evaluations, one would need to develop and validate prototypical deployment examples apart from testing implicit processes under laboratory conditions by including specific reaction-time measures, for instance. As indicated by the two examples for NT, benefit estimation depends on the specificities of the example: while the high attribution of usefulness (represented by the item ‘beneficial’) and the potential to generate money that was attributed to NT in the implicit task was confirmed in the deployment example for environmental benefits (nano-fabricated solar cells), it could (understandably) not be confirmed in the deployment example for benefits for humans (nano sport cloths).

Another potential limitation of our study concerns a potential bias in the preselection of items. Preselection was performed by a broad literature review and was complemented until the authors were not able to add any new items. One could criticize that, in the resulting list of positive and negative items, there were several negative items with moralistic connotations (megalomaniac, soulless, presumptuous, and reprehensible), which might be less frequently selected in general than the more factual positive items. However, our observation that negative items were selected less frequently than positive ones was also true for the “factual” items ‘risky,’ ‘uncontrollable,’ ‘ecologically harmful,’ and ‘dangerous.’ Further, if the number of negative items was reduced by those with a moralistic connotation, one would expect the more factual items to be selected even more vigorously. Finally, even for AGT, the items ‘risky’ and ‘dangerous’ were selected less frequently than the positively connoted items ‘beneficial’ and ‘sophisticated,’ making the argument of generally positive technology profiles even stronger.

## Conclusion

In this study, we aimed at collecting data to investigate the image of the two emerging technologies NT and SB. To this end, we investigated spontaneous associations, explicit harm-benefit estimations and questions about regulation and compared the assessment of NT and SB with those of the more established technologies CT and AGT.

We observed that overall, the implicit technology profile of SB and NT is positive and that the investigated technologies enjoy different esteem, with synthetic biology and nanotechnology both showing a more “exciting” image that is not necessarily benefit-related. Even though the image of nanotechnology was demonstrated to be more pronounced and uniform between groups it was also more heterogeneous across tasks while agricultural biotechnology remains contested. The positive outcome could be reproduced in the explicit harm-benefit assessment with benefits generally surpassing harm. Surprisingly, NT was revealed to be associated even more positively than the widely used CT. The results gained from the implicit association task play a key role for the image of a technology that cannot be grasped by traditional harm-benefit evaluations. Moreover, the implicit task facilitates a direct comparison of the perceptions toward the four technologies. Such a comparison is more difficult in explicit harm-benefit evaluations because the assessment depends to a large degree on the specific deployment examples presented to the study participants. Accordingly, more studies should investigate implicit technology perception to help fostering the generation of nuanced technology profiles by collecting more representative data.

The inter-technological comparison revealed that the quantitative aspects of the technology profiles of SB in many respects are intermediate between NT and AGT, while, qualitatively, SB and NT share a high degree of similarity. Hence, there are no indications for a general mistrust or opposition against this technology, which is generally evaluated more positively than AGT.

A comparison between the responses of the implicit association task and the explicit harm-benefit evaluation or explicit views on how the technologies should be regulated revealed that spontaneous associations might be revised upon confrontation with specific examples. Particularly, the harm potential of NT and the benefit potential of SB were estimated to be higher when concrete examples were evaluated. Moreover, requirements for regulation were more in accordance with NT’s particularly positive implicit associations rather than its elevated explicit harm attribution.

The present study finally provides a basis to longitudinally and prospectively investigate the image of emerging technologies.

## Author Contributions

CI and AD-Z constructed the survey, CI analyzed the data and CI and AD-Z wrote the paper. NB-A provided valuable feedback and substantially contributed to this work during the process of writing. CI furthermore confirms that he has final responsibility for the decision to submit for publication.

## Conflict of Interest Statement

The authors declare that the research was conducted in the absence of any commercial or financial relationships that could be construed as a potential conflict of interest.

## Footnotes

1 http://www.qualtrics.com/
