# Supplementary material for: Image of Synthetic Biology and Nanotechnology: A Survey among University Students
Source: Front Genet. 2017 Sep 20;8:122. doi: 10.3389/fgene.2017.00122 (PMC5611450; doi:10.3389/fgene.2017.00122)
Supplement: Supplementary file 1 [file Presentation_1.pdf]

Supplementary Materials

Table S1: Items (German original and English translation; no back-translation performed) used as stimulus material for association task.

German	English
faszinierend	fascinating
sollte man fördern	something one should encourage
fortschrittlich	progressive
generiert geld	generates money
gut	good
komplex	sophisticated
kontrollierbar	controllable
nützlich	beneficial
ökologisch/umweltbewusst	ecological, environmentally aware
vielseitig	versatile
vielversprechend	promising
gefährlich	dangerous
grössenwahnsinnig	megalomaniac
riskant	risky
schlecht	bad
seelenlos	soulless
überheblich	presumtious
umweltschädlich	ecologically harmful
unheimlich	scary
unkontrollierbar	uncontrollable
sollte man verbieten	something one should prohibit
verwerflich	reprehensible

Table S2: Item matrices for performing correlational analyses that formed the basis for investigating qualitative differences. A: Basic item matrices of differences of normalized selection frequencies among all positive items and for all technologies investigated. B: Basic item matrices of differences of normalized selection frequencies among all positive items for investigating effect of academic background. C: Basic item matrices of differences of normalized selection frequencies among all positive items for investigating effect of gender.

A)

CT	fascinating	sth one should encourage	progressive	generates money	good	sophisticated	controllable	beneficial	ecological	versatile	promising
**fascinating**	0	0.077341	0.313433	0.370421	0.126866	0.256446	0.050204	0.502036	-0.1689276	0.341249	0.113298
**sth one should encourage**	-0.077341	0	0.236092	0.29308	0.049525	0.179105	-0.027137	0.424695	-0.2462686	0.263908	0.035957
**progressive**	-0.313433	-0.236092	0	0.056988	-0.186567	-0.056987	-0.263229	0.188603	-0.4823606	0.027816	-0.200135
**generates money**	-0.370421	-0.29308	-0.056988	0	-0.243555	-0.113975	-0.320217	0.131615	-0.5393486	-0.029172	-0.257123
**good**	-0.126866	-0.049525	0.186567	0.243555	0	0.12958	-0.076662	0.37517	-0.2957936	0.214383	-0.013568
**sophisticated**	-0.256446	-0.179105	0.056987	0.113975	-0.12958	0	-0.206242	0.24559	-0.4253736	0.084803	-0.143148
**controllable**	-0.050204	0.027137	0.263229	0.320217	0.076662	0.206242	0	0.451832	-0.2191316	0.291045	0.063094
**beneficial**	-0.502036	-0.424695	-0.188603	-0.131615	-0.37517	-0.24559	-0.451832	0	-0.6709636	-0.160787	-0.388738
**ecological**	0.1689276	0.2462686	0.4823606	0.5393486	0.2957936	0.4253736	0.2191316	0.6709636	0	0.5101766	0.2822256
**versatile**	-0.341249	-0.263908	-0.027816	0.029172	-0.214383	-0.084803	-0.291045	0.160787	-0.5101766	0	-0.227951
**promising**	-0.113298	-0.035957	0.200135	0.257123	0.013568	0.143148	-0.063094	0.388738	-0.2822256	0.227951	0

AGT	fascinating	sth one should encourage	progressive	generates money	good	sophisticated	controllable	beneficial	ecological	versatile	promising
**fascinating**	0	-0.067164	0.078019	0.007463	-0.169606	0.148575	-0.261194	0.171642	-0.0346	-0.028494	0.093623
**sth one should encourage**	0.067164	0	0.145183	0.074627	-0.102442	0.215739	-0.19403	0.238806	0.032564	0.03867	0.160787
**progressive**	-0.078019	-0.145183	0	-0.070556	-0.247625	0.070556	-0.339213	0.093623	-0.112619	-0.106513	0.015604

**generates money**	-0.007463	-0.074627	0.070556	0	-0.177069	0.141112	-0.268657	0.164179	-0.042063	-0.035957	0.08616
**good**	0.169606	0.102442	0.247625	0.177069	0	0.318181	-0.091588	0.341248	0.135006	0.141112	0.263229
**sophisticated**	-0.148575	-0.215739	-0.070556	-0.141112	-0.318181	0	-0.409769	0.023067	-0.183175	-0.177069	-0.054952
**controllable**	0.261194	0.19403	0.339213	0.268657	0.091588	0.409769	0	0.432836	0.226594	0.2327	0.354817
**beneficial**	-0.171642	-0.238806	-0.093623	-0.164179	-0.341248	-0.023067	-0.432836	0	-0.206242	-0.200136	-0.078019
**ecological**	0.0346	-0.032564	0.112619	0.042063	-0.135006	0.183175	-0.226594	0.206242	0	0.006106	0.128223
**versatile**	0.028494	-0.03867	0.106513	0.035957	-0.141112	0.177069	-0.2327	0.200136	-0.006106	0	0.122117
**promising**	-0.093623	-0.160787	-0.015604	-0.08616	-0.263229	0.054952	-0.354817	0.078019	-0.128223	-0.122117	0

**NT**	**fascinating**	**sth one should encourage**	**progressive**	**generates money**	**good**	**sophisticated**	**controllable**	**beneficial**	**ecological**	**versatile**	**promising**
**fascinating**	0	-0.189959	0.046133	-0.261194	-0.386024	0.004071	-0.463365	-0.052238	-0.6105831	-0.08616	-0.014247
**sth one should encourage**	0.189959	0	0.236092	-0.071235	-0.196065	0.19403	-0.273406	0.137721	-0.4206241	0.103799	0.175712
**progressive**	-0.046133	-0.236092	0	-0.307327	-0.432157	-0.042062	-0.509498	-0.098371	-0.6567161	-0.132293	-0.06038
**generates money**	0.261194	0.071235	0.307327	0	-0.12483	0.265265	-0.202171	0.208956	-0.3493891	0.175034	0.246947
**good**	0.386024	0.196065	0.432157	0.12483	0	0.390095	-0.077341	0.333786	-0.2245591	0.299864	0.371777
**sophisticated**	-0.004071	-0.19403	0.042062	-0.265265	-0.390095	0	-0.467436	-0.056309	-0.6146541	-0.090231	-0.018318
**controllable**	0.463365	0.273406	0.509498	0.202171	0.077341	0.467436	0	0.411127	-0.1472181	0.377205	0.449118
**beneficial**	0.052238	-0.137721	0.098371	-0.208956	-0.333786	0.056309	-0.411127	0	-0.5583451	-0.033922	0.037991
**ecological**	0.6105831	0.4206241	0.6567161	0.3493891	0.2245591	0.6146541	0.1472181	0.5583451	0	0.5244231	0.5963361
**versatile**	0.08616	-0.103799	0.132293	-0.175034	-0.299864	0.090231	-0.377205	0.033922	-0.5244231	0	0.071913
**promising**	0.014247	-0.175712	0.06038	-0.246947	-0.371777	0.018318	-0.449118	-0.037991	-0.5963361	-0.071913	0

SB	fascinating	sth one should encourage	progressive	generates money	good	sophisticated	controllable	beneficial	ecological	versatile	promising
**fascinating**	0	-0.240163	0.004071	-0.283582	-0.349389	0.124831	-0.364993	-0.054952	-0.451153	-0.057666	-0.065807
**sth one should encourage**	0.240163	0	0.244234	-0.043419	-0.109226	0.364994	-0.12483	0.185211	-0.21099	0.182497	0.174356
**progressive**	-0.004071	-0.244234	0	-0.287653	-0.35346	0.12076	-0.369064	-0.059023	-0.455224	-0.061737	-0.069878
**generates money**	0.283582	0.043419	0.287653	0	-0.065807	0.408413	-0.081411	0.22863	-0.167571	0.225916	0.217775
**good**	0.349389	0.109226	0.35346	0.065807	0	0.47422	-0.015604	0.294437	-0.101764	0.291723	0.283582
**sophisticated**	-0.124831	-0.364994	-0.12076	-0.408413	-0.47422	0	-0.489824	-0.179783	-0.575984	-0.182497	-0.190638
**controllable**	0.364993	0.12483	0.369064	0.081411	0.015604	0.489824	0	0.310041	-0.08616	0.307327	0.299186
**beneficial**	0.054952	-0.185211	0.059023	-0.22863	-0.294437	0.179783	-0.310041	0	-0.396201	-0.002714	-0.010855
**ecological**	0.451153	0.21099	0.455224	0.167571	0.101764	0.575984	0.08616	0.396201	0	0.393487	0.385346
**versatile**	0.057666	-0.182497	0.061737	-0.225916	-0.291723	0.182497	-0.307327	0.002714	-0.393487	0	-0.008141
**promising**	0.065807	-0.174356	0.069878	-0.217775	-0.283582	0.190638	-0.299186	0.010855	-0.385346	0.008141	0

B)

CT-nat	fascinating	sth one should encourage	progressive	generates money	good	sophisticated	controllable	beneficial	ecological	versatile	promising
**fascinating**	0										
**sth one should encourage**	-0.104575	0									
**progressive**	-0.333333	-0.228758	0								
**generates money**	-0.413072	-0.308497	-0.079739	0							
**good**	-0.133333	-0.028758	0.2	0.279739	0						
**sophisticated**	-0.27451	-0.169935	0.058823	0.138562	-0.141177	0					
**controllable**	-0.071895	0.03268	0.261438	0.341177	0.061438	0.202615	0				

**beneficial**	-0.537255	-0.43268	-0.203922	-0.124183	-0.403922	-0.262745	-0.46536	0			
**ecological**	0.128105	0.23268	0.461438	0.541177	0.261438	0.402615	0.2	0.66536	0		
**versatile**	-0.392157	-0.287582	-0.058824	0.020915	-0.258824	-0.117647	-0.320262	0.145098	-0.520262	0	
**promising**	-0.146405	-0.04183	0.186928	0.266667	-0.013072	0.128105	-0.07451	0.39085	-0.27451	0.245752	0

**CT-hum**	**fascinating**	**sth one should encourage**	**progressive**	**generates money**	**good**	**sophisticated**	**controllable**	**beneficial**	**ecological**	**versatile**	**promising**
**fascinating**	0										
**sth one should encourage**	0.00641	0									
**progressive**	-0.294872	-0.301282	0								
**generates money**	-0.326923	-0.333333	-0.032051	0							
**good**	-0.025641	-0.032051	0.269231	0.301282	0						
**sophisticated**	-0.211538	-0.217948	0.083334	0.115385	-0.185897	0					
**controllable**	0.051282	0.044872	0.346154	0.378205	0.076923	0.26282	0				
**beneficial**	-0.403846	-0.410256	-0.108974	-0.076923	-0.378205	-0.192308	-0.455128	0			
**ecological**	0.211539	0.205129	0.506411	0.538462	0.23718	0.423077	0.160257	0.615385	0		
**versatile**	-0.25	-0.25641	0.044872	0.076923	-0.224359	-0.038462	-0.301282	0.153846	-0.461539	0	
**promising**	-0.064103	-0.070513	0.230769	0.26282	-0.038462	0.147435	-0.115385	0.339743	-0.275642	0.185897	0

**AGT-nat**	**fascinating**	**sth one should encourage**	**progressive**	**generates money**	**good**	**sophisticated**	**controllable**	**beneficial**	**ecological**	**versatile**	**promising**
**fascinating**	0										
**sth one should encourage**	0.045751	0									
**progressive**	-0.058824	-0.104575	0								

**generates money**	0.024836	-0.020915	0.08366	0							
**good**	0.180392	0.134641	0.239216	0.155556	0						
**sophisticated**	-0.126798	-0.172549	-0.067974	-0.151634	-0.30719	0					
**controllable**	0.28366	0.237909	0.342484	0.258824	0.103268	0.410458	0				
**beneficial**	-0.156863	-0.202614	-0.098039	-0.181699	-0.337255	-0.030065	-0.440523	0			
**ecological**	0.048366	0.002615	0.10719	0.02353	-0.132026	0.175164	-0.235294	0.205229	0		
**versatile**	0.044444	-0.001307	0.103268	0.019608	-0.135948	0.171242	-0.239216	0.201307	-0.003922	0	
**promising**	-0.108497	-0.154248	-0.049673	-0.133333	-0.288889	0.018301	-0.392157	0.048366	-0.156863	-0.152941	0

**AGT-hum**	**fascinating**	**sth one should encourage**	**progressive**	**generates money**	**good**	**sophisticated**	**controllable**	**beneficial**	**ecological**	**versatile**	**promising**
**fascinating**	0										
**sth one should encourage**	0.044872	0									
**progressive**	-0.096154	-0.141026	0								
**generates money**	-0.108974	-0.153846	-0.01282	0							
**good**	0.128205	0.083333	0.224359	0.237179	0						
**sophisticated**	-0.237179	-0.282051	-0.141025	-0.128205	-0.365384	0					
**controllable**	0.173077	0.128205	0.269231	0.282051	0.044872	0.410256	0				
**beneficial**	-0.192308	-0.23718	-0.096154	-0.083334	-0.320513	0.044871	-0.365385	0			
**ecological**	-0.044872	-0.089744	0.051282	0.064102	-0.173077	0.192307	-0.217949	0.147436	0		
**versatile**	-0.012821	-0.057693	0.083333	0.096153	-0.141026	0.224358	-0.185898	0.179487	0.032051	0	
**promising**	-0.096154	-0.141026	0	0.01282	-0.224359	0.141025	-0.269231	0.096154	-0.051282	-0.083333	0

**NT-nat**	**fascinating**	**sth one should encourage**	**progressive**	**generates money**	**good**	**sophisticated**	**controllable**	**beneficial**	**ecological**	**versatile**	**promising**

**fascinating**	0										
**sth one should encourage**	0.188235	0									
**progressive**	-0.037909	-0.226144	0								
**generates money**	0.28366	0.095425	0.321569	0							
**good**	0.402614	0.214379	0.440523	0.118954	0						
**sophisticated**	0.005228	-0.183007	0.043137	-0.278432	-0.397386	0					
**controllable**	0.457516	0.269281	0.495425	0.173856	0.054902	0.452288	0				
**beneficial**	0.035294	-0.152941	0.073203	-0.248366	-0.36732	0.030066	-0.422222	0			
**ecological**	0.614379	0.426144	0.652288	0.330719	0.211765	0.609151	0.156863	0.579085	0		
**versatile**	0.084967	-0.103268	0.122876	-0.198693	-0.317647	0.079739	-0.372549	0.049673	-0.529412	0	
**promising**	-0.006536	-0.194771	0.031373	-0.290196	-0.40915	-0.011764	-0.464052	-0.04183	-0.620915	-0.091503	0

**NT-hum**	**fascinating**	**sth one should encourage**	**progressive**	**generates money**	**good**	**sophisticated**	**controllable**	**beneficial**	**ecological**	**versatile**	**promising**
**fascinating**	0										
**sth one should encourage**	0.224359	0									
**progressive**	-0.064103	-0.288462	0								
**generates money**	0.173077	-0.051282	0.23718	0							
**good**	0.371795	0.147436	0.435898	0.198718	0						
**sophisticated**	-0.083333	-0.307692	-0.01923	-0.25641	-0.455128	0					
**controllable**	0.442308	0.217949	0.506411	0.269231	0.070513	0.525641	0				
**beneficial**	0.121795	-0.102564	0.185898	-0.051282	-0.25	0.205128	-0.320513	0			
**ecological**	0.512821	0.288462	0.576924	0.339744	0.141026	0.596154	0.070513	0.391026	0		
**versatile**	0.070513	-0.153846	0.134616	-0.102564	-0.301282	0.153846	-0.371795	-0.051282	-0.442308	0	
**promising**	-0.00641	-0.230769	0.057693	-0.179487	-0.378205	0.076923	-0.448718	-0.128205	-0.519231	-0.076923	0

SB-nat	fascinating	sth one should encourage	progressive	generates money	good	sophisticated	controllable	beneficial	ecological	versatile	promising
fascinating	0										
sth one should encourage	0.219608	0									
progressive	-0.005228	-0.224836	0								
generates money	0.329412	0.109804	0.33464	0							
good	0.354249	0.134641	0.359477	0.024837	0						
sophisticated	-0.111111	-0.330719	-0.105883	-0.440523	-0.46536	0					
controllable	0.371242	0.151634	0.37647	0.04183	0.016993	0.482353	0				
beneficial	0.057517	-0.162091	0.062745	-0.271895	-0.296732	0.168628	-0.313725	0			
ecological	0.4718958	0.2522878	0.4771238	0.1424838	0.1176468	0.5830068	0.1006538	0.4143788	0		
versatile	0.050981	-0.168627	0.056209	-0.278431	-0.303268	0.162092	-0.320261	-0.006536	-0.4209148	0	
promising	0.062745	-0.156863	0.067973	-0.266667	-0.291504	0.173856	-0.308497	0.005228	-0.4091508	0.011764	0

SB-hum	fascinating	sth one should encourage	progressive	generates money	good	sophisticated	controllable	beneficial	ecological	versatile	promising
fascinating	0										
sth one should encourage	0.301282	0									
progressive	0.032052	-0.26923	0								
generates money	0.134616	-0.166666	0.102564	0							
good	0.3589747	0.0576927	0.3269227	0.2243587	0						
sophisticated	-0.224359	-0.525641	-0.256411	-0.358975	-0.5833337	0					
controllable	0.339744	0.038462	0.307692	0.205128	-0.0192307	0.564103	0				
beneficial	0.128205	-0.173077	0.096153	-0.006411	-0.2307697	0.352564	-0.211539	0			
ecological	0.4230772	0.1217952	0.3910252	0.2884612	0.0641025	0.6474362	0.0833332	0.2948722	0		

**versatile**	0.083334	-0.217948	0.051282	-0.051282	-0.2756407	0.307693	-0.25641	-0.044871	-0.3397432	0	
**promising**	0.102564	-0.198718	0.070512	-0.032052	-0.2564107	0.326923	-0.23718	-0.025641	-0.3205132	0.01923	0

C)

**CT-Females**	**fascinating**	**sth one should encourage**	**progressive**	**generates money**	**good**	**sophisticated**	**controllable**	**beneficial**	**ecological**	**versatile**	**promising**
**fascinating**	0										
**sth one should encourage**	-0.08541973	0									
**progressive**	-0.33136966	-0.24594993	0								
**generates money**	-0.35051546	-0.26509573	-0.0191458	0							
**good**	-0.09572901	-0.01030928	0.23564065	0.25478645	0						
**sophisticated**	-0.23416789	-0.14874816	0.09720177	0.11634757	-0.13843888	0					
**controllable**	-0.01178203	0.0736377	0.31958763	0.33873343	0.08394698	0.22238586	0				
**beneficial**	-0.51399116	-0.42857143	-0.1826215	-0.1634757	-0.41826215	-0.27982327	-0.50220913	0			
**ecological**	0.14727541	0.23269514	0.47864507	0.49779087	0.24300442	0.3814433	0.15905744	0.66126657	0		
**versatile**	-0.35640648	-0.27098675	-0.02503682	-0.00589102	-0.26067747	-0.12223859	-0.34462445	0.15758468	-0.50368189	0	
**promising**	-0.13254786	-0.04712813	0.1988218	0.2179676	-0.03681885	0.10162003	-0.12076583	0.3814433	-0.27982327	0.22385862	0

**CT-Males**	**fascinating**	**sth one should encourage**	**progressive**	**generates money**	**good**	**sophisticated**	**controllable**	**beneficial**	**ecological**	**versatile**	**promising**
**fascinating**	0										
**sth one should encourage**	-0.07044025	0									
**progressive**	-0.29811321	-0.22767296	0								
**generates money**	-0.38742138	-0.31698113	-0.08930818	0							

**good**	-0.15345912	-0.08301887	0.14465409	0.23396226	0						
**sophisticated**	-0.2754717	-0.20503145	0.02264151	0.11194969	-0.12201258	0					
**controllable**	-0.08301887	-0.01257862	0.21509434	0.30440252	0.07044025	0.19245283	0				
**beneficial**	-0.4918239	-0.42138365	-0.19371069	-0.10440252	-0.33836478	-0.2163522	-0.40880503	0			
**ecological**	0.18742138	0.25786164	0.48553459	0.57484277	0.3408805	0.46289308	0.27044025	0.67924528	0		
**versatile**	-0.32830189	-0.25786164	-0.03018868	0.0591195	-0.17484277	-0.05283019	-0.24528302	0.16352201	-0.51572327	0	
**promising**	-0.09685535	-0.02641509	0.20125786	0.29056604	0.05660377	0.17861635	-0.01383648	0.39496855	-0.28427673	0.23144654	0

AGT-Females	fascinating	sth one should encourage	progressive	generates money	good	sophisticated	controllable	beneficial	ecological	versatile	promising
**fascinating**	0										
**sth one should encourage**	0.09278351	0									
**progressive**	-0.06774669	-0.16053019	0								
**generates money**	-0.00736377	-0.10014728	0.06038292	0							
**good**	0.19145803	0.09867452	0.25920471	0.1988218	0						
**sophisticated**	-0.15905744	-0.25184094	-0.09131075	-0.15169367	-0.35051546	0					
**controllable**	0.27098675	0.17820324	0.33873343	0.27835052	0.07952872	0.43004418	0				
**beneficial**	-0.16494845	-0.25773196	-0.09720177	-0.15758468	-0.35640648	-0.00589102	-0.4359352	0			
**ecological**	0.01767305	-0.07511046	0.08541973	0.02503682	-0.17378498	0.17673049	-0.2533137	0.1826215	0		
**versatile**	0.00736377	-0.08541973	0.07511046	0.01472754	-0.18409426	0.16642121	-0.26362297	0.17231222	-0.01030928	0	
**promising**	-0.09131075	-0.18409426	-0.02356406	-0.08394698	-0.28276878	0.06774669	-0.3622975	0.0736377	-0.1089838	-0.09867452	0

AGT-Males	fascinating	sth one should encourage	progressive	generates money	good	sophisticated	controllable	beneficial	ecological	versatile	promising
**fascinating**	0										

**sth one should encourage**	0.04528302	0									
**progressive**	-0.08679245	-0.13207547	0								
**generates money**	-0.00754717	-0.05283019	0.07924528	0							
**good**	0.1509434	0.10566038	0.23773585	0.15849057	0						
**sophisticated**	-0.13962264	-0.18490566	-0.05283019	-0.13207547	-0.29056604	0					
**controllable**	0.25283019	0.20754717	0.33962264	0.26037736	0.10188679	0.39245283	0				
**beneficial**	-0.17735849	-0.22264151	-0.09056604	-0.16981132	-0.32830189	-0.03773585	-0.43018868	0			
**ecological**	0.0490566	0.00377358	0.13584906	0.05660377	-0.10188679	0.18867925	-0.20377358	0.22641509	0		
**versatile**	0.04654088	0.00125786	0.13333333	0.05408805	-0.10440252	0.18616352	-0.20628931	0.22389937	-0.00251572	0	
**promising**	-0.09559748	-0.1408805	-0.00880503	-0.08805031	-0.24654088	0.04402516	-0.34842767	0.08176101	-0.14465409	-0.14213836	0

**NT-Females**	**fascinating**	**sth one should encourage**	**progressive**	**generates money**	**good**	**sophisticated**	**controllable**	**beneficial**	**ecological**	**versatile**	**promising**
**fascinating**	0										
**sth one should encourage**	0.2533137	0									
**progressive**	-0.00147275	-0.25478645	0								
**generates money**	0.28865979	0.0353461	0.29013255	0							
**good**	0.45066274	0.19734904	0.45213549	0.16200295	0						
**sophisticated**	0.01472754	-0.23858616	0.01620029	-0.27393225	-0.4359352	0					
**controllable**	0.50220913	0.24889543	0.50368189	0.21354934	0.05154639	0.48748159	0				
**beneficial**	0.12076583	-0.13254786	0.12223859	-0.16789396	-0.32989691	0.10603829	-0.3814433	0			
**ecological**	0.62150221	0.36818851	0.62297496	0.33284242	0.17083947	0.60677467	0.11929308	0.50073638	0		
**versatile**	0.10456554	-0.14874816	0.10603829	-0.18409426	-0.3460972	0.089838	-0.39764359	-0.01620029	-0.51693667	0	
**promising**	0.03829161	-0.21502209	0.03976436	-0.25036819	-0.41237113	0.02356406	-0.46391753	-0.08247423	-0.5832106	-0.06627393	0

NT-Males	fascinating	sth one should encourage	progressive	generates money	good	sophisticated	controllable	beneficial	ecological	versatile	promising
fascinating	0										
sth one should encourage	0.13584906	0									
progressive	-0.08427673	-0.22012579	0								
generates money	0.23773585	0.10188679	0.32201258	0							
good	0.33081761	0.19496855	0.41509434	0.09308176	0						
sophisticated	-0.02012579	-0.15597484	0.06415094	-0.25786164	-0.3509434	0					
controllable	0.43018868	0.29433962	0.51446541	0.19245283	0.09937107	0.45031447	0				
beneficial	-0.00628931	-0.14213836	0.07798742	-0.24402516	-0.33710692	0.01383648	-0.43647799	0			
ecological	0.60125786	0.46540881	0.68553459	0.36352201	0.27044025	0.62138365	0.17106918	0.60754717	0		
versatile	0.07044025	-0.06540881	0.15471698	-0.1672956	-0.26037736	0.09056604	-0.35974843	0.07672956	-0.53081761	0	
promising	-0.00628931	-0.14213836	0.07798742	-0.24402516	-0.33710692	0.01383648	-0.43647799	0	-0.60754717	-0.07672956	0

SB-Females	fascinating	sth one should encourage	progressive	generates money	good	sophisticated	controllable	beneficial	ecological	versatile	promising
fascinating	0										
sth one should encourage	0.24742268	0									
progressive	-0.01178203	-0.25920471	0								
generates money	0.25478645	0.00736377	0.26656848	0							
good	0.35935199	0.11192931	0.37113402	0.10456554	0						
sophisticated	-0.15316642	-0.4005891	-0.14138439	-0.40795287	-0.51251841	0					
controllable	0.34462445	0.09720177	0.35640648	0.089838	-0.01472754	0.49779087	0				
beneficial	0.02209131	-0.22533137	0.03387334	-0.23269514	-0.33726068	0.17525773	-0.32253314	0			
ecological	0.43004418	0.1826215	0.44182622	0.17525773	0.07069219	0.5832106	0.08541973	0.40795287	0		

**versatile**	0.02356406	-0.22385862	0.0353461	-0.23122239	-0.33578792	0.17673049	-0.32106038	0.00147275	-0.40648012	0	
**promising**	0.0544919	-0.19293078	0.06627393	-0.20029455	-0.30486009	0.20765832	-0.29013255	0.03240059	-0.37555228	0.03092784	0

**SB-Males**	**fascinating**	**sth one should encourage**	**progressive**	**generates money**	**good**	**sophisticated**	**controllable**	**beneficial**	**ecological**	**versatile**	**promising**
**fascinating**	0										
**sth one should encourage**	0.23396226	0									
**progressive**	0.00251572	-0.23144654	0								
**generates money**	0.3081761	0.07421384	0.30566038	0							
**good**	0.3408805	0.10691824	0.33836478	0.0327044	0						
**sophisticated**	-0.10062893	-0.33459119	-0.10314465	-0.40880503	-0.44150943	0					
**controllable**	0.38238994	0.14842767	0.37987421	0.07421384	0.04150943	0.48301887	0				
**beneficial**	0.08301887	-0.1509434	0.08050314	-0.22515723	-0.25786164	0.1836478	-0.29937107	0			
**ecological**	0.46918239	0.23522013	0.46666667	0.16100629	0.12830189	0.56981132	0.08679245	0.38616352	0		
**versatile**	0.08679245	-0.14716981	0.08427673	-0.22138365	-0.25408805	0.18742138	-0.29559748	0.00377358	-0.38238994	0	
**promising**	0.0754717	-0.15849057	0.07295597	-0.2327044	-0.26540881	0.17610063	-0.30691824	-0.00754717	-0.39371069	-0.01132075	0

Table S3: Statements (German original and English translation; no back-translation) used as stimulus material for harm & benefit estimation.

**Harm (D)/ Benefit (B)**	**Technology / Condition**	**Description German** Wie evaluieren Sie das Schadenspotenzial / den Nutzen der folgenden Anwendungen…	**Description English** How do you evaluate the harm-potential / the benefit-potential of the following applications…
D	Nanotechnology / Environment	… Das Schadenspotential von Nanopartikeln (sehr kleine Teilchen), die aus dem Zerfall von benutztem Nano-Sonnenschutzmittel ins Wasser und in die Umwelt gelangen.	… The harm potential of sunscreen-nanoparticles escaping into the environment.
D	Nanotechnology / Human	… Das Schadenspotential von Nanopartikeln im Verpackungsmaterial von Nahrungsmitteln für den Menschen.	… The harm potential of nanoparticles in food packaging for humans.
D	Synthetic biology / Environment	… Das Schadenspotential von Bakterien, die über synthetisch-biologische Verfahren verändert wurden und als Indikatoren zur Schwermetallerkennung in den Boden/die Umwelt gegeben wurden.	… The harm potential of synthetic biologically modified bacteria in form of bio-indicators for heavy metals released into the environment.
D	Synthetic biology / Human	... Das Schadenspotential von synthetisch-biologischen Implantaten, die Krebszellen im Menschen erkennen.	… The harm potential of synthetic biology in the context of closed loop systems for the recognition of cancer cells in humans.
D	Agricultural Biotechnology / Environment	… Das Schadenspotential für die Umwelt von gentechnisch verändertem Mais, der aktiv ein Gift zur Bekämpfung von Schädlingen (Parasiten) herstellen kann.	… The harm potential of bio-technologically modified maize with intrinsic ability to defend against vermin.
D	Agricultural Biotechnology / Human	… Das Schadenspotential von gentechnisch veränderten Lebensmitteln (wie Sojaprodukten) für den Menschen.	… The harm potential of bio-technologically modified food products (e.g. soy products) for humans.
B	Nanotechnology / Environment	… Der Nutzen für die Umwelt von Nanomaterialien für effizientere Solarzellen.	… The benefit potential of nano-technologically produced solar cells for the environment.
B	Nanotechnology / Human	… Der Nutzen von Nano-Kleidung aus atmungsaktiven Kunstfasern für den Menschen.	… The benefit potential of nano-technologically fabricated sports-clothing for humans.
B	Synthetic biology / Environment	… Der Nutzen für die Umwelt von Ölteppich-abbauenden Bakterien welche über synthetisch-biologischen Verfahren entwickelt wurden.	… The benefit potential of synthetically modified oil-slick-degrading bacteria for the environment.
B	Synthetic biology / Human	… Der Nutzen für den Menschen von neuen Antibiotika, hergestellt über synthetisch-biologischen Verfahren.	… The benefit potential of synthetic biologically fabricated antibiotics for humans.
B	Agricultural Biotechnology / Environment	… Der Nutzen vom Anbau von gentechnisch veränderten Nutzpflanzen für die Umwelt, um die Menge von verwendeten Insektiziden / Herbiziden zu reduzieren und damit die Umweltbelastung zu minimieren.	… The benefit potential of bio-technologically modified maize to reduce herbicides/insecticides and hence minimize environmental pollution.
B	Agricultural Biotechnology / Human	… Der Nutzen von Reis für den Menschen, der durch gentechnische Verfahren eine deutlich erhöhte Menge	… The benefit potential of bio-technologically modified rice with

		an Beta-Carotin (Provitamin A) aufweist.	increased levels of pro-vitamin A for humans.

Table S4: Ordinal logistic regression models testing academic background and gender as determinants of technology assessment (danger potential)

Ordinal logistic regression models									
	Nano-Sunscreen			Nano-Packing			SB-Bioindicator		
	Estimate	SE	Odds	Estimate	SE	Odds	Estimate	SE	Odds
Treshhold 1	-1.960***	.130		-1.764***	.121		-.723***	.099	
Treshhold 2	-.166	.087		-.103	.095		.712***	.099	
Treshhold 3	1.205***	.093		1.305***	.105		1.840***	.116	
Gender	.551***	.102	1.73	.657***	.122	1.93	.417***	.123	1.52
Humanities and social sciences	.428**	.165	1.53	.147	.163	1.16	.767***	.169	2.15
Nagelkerke R^2		0.039			0.036			0.042	
Parallel lines		Yes			Yes			Yes	

*p < .05; **p < .01; ***p < .001.

Note. Odds ratios presented above, as well as in subsequent ordinal regression tables, were computed as exp(b).

Table S4 cont. Ordinal logistic regression models testing academic background and gender as determinants of technology assessment (danger potential)

Ordinal logistic regression models									
	SB-ClosedLoop			AGT-Maize			AGT-Soy		
	Estimate	SE	Odds	Estimate	SE	Odds	Estimate	SE	Odds
Treshhold 1	-.810***	.102		-1.605***	.116		.043	.097	
Treshhold 2	1.084***	.105		-.007	.095		1.426**	.109	
Treshhold 3	2.540***	.141		1.0883***	.102		2.605***	.136	
Gender	.330*	.126	1.39	.475***	.122	1.61	.653***	.125	1.92
Humanities & Social Sciences	.476**	.170	1.61	.486**	.165	1.63	1.139***	.166	3.12
Nagelkerke R^2		0.019			0.030			0.087	
Parallel lines		Yes			Yes			Yes	

*p < .05; **p < .01; ***p < .001.

Table S5. Ordinal logistic regression models testing academic background and gender as determinants of technology assessment (benefit potential)

Ordinal logistic regression models									
	Nano-Clothing			Nano-Solar			SB-Oil slick		
	Estimate	SE	Odds	Estimate	SE	Odds	Estimate	SE	Odds
Treshhold 1	-2.013***	.124		-4.107***	.274		-4.029***	.272	
Treshhold 2	-.258**	.096		-2.354***	.144		-2.094***	.134	
Treshhold 3	.898***	.101		-.869***	.110		-.680***	.106	
Gender	-.049	.123	0.95	-.095	.145	0.91	.170	.142	1.19
Humanities & Social Sciences	-.170	.165	0.84	-.045	.193	0.96	-.377*	.185	0.69
Nagelkerke R^2		0.002			0.001			0.007	
Parallel lines		Yes			Yes			Yes	

*p < .05; **p < .01; ***p < .001.

Table S5 cont. Ordinal logistic regression models testing academic background and gender as determinants of technology assessment (benefit potential)

Ordinal logistic regression models									
	SB-Antibiotics			AGT-Rice			AGT-Insecticide		
	Estimate	SE	Odds	Estimate	SE	Odds	Estimate	SE	Odds
Treshhold 1	-3.701***	.218		-2.313***	.134		-2.527***	.141	
Treshhold 2	-1.973***	.128		-.693***	.100		-1.175***	.106	
Treshhold 3	-.861***	.109		.292**	.098		.236*	.098	
Gender	-.070	.142	0.93	-.012	.125	0.99	-.124	.125	0.88
Humanities & Social Sciences	-.786***	.177	0.46	-.916***	.167	0.40	-.971***	.167	0.38
Nagelkerke R^2		0.028			0.038			0.043	
Parallel lines		No			Yes			Yes	

*p < .05; **p < .01; ***p < .001.

Table S5 cont. Multinominal logistic regression models testing academic background and gender as determinants of technology assessment (benefit potential).

	SB-Antibiotics		
	Estimate	SE	Odds
Small			
Intercept	-3.115***	.293	
Hum. & Soc. Sci.	.322	.560	1.38
Gender	-.062	.402	0.94
Small-Medium			
Intercept	-1.924***	.166	
Hum. & Soc. Sci.	.788**	.271	2.20
Gender	.111	.217	1.12
Medium-High			
Intercept	-1.528	.139	
Hum. & Soc. Sci.	1.143***	.214	3.14
Gender	.120	.180	1.11
Nagelkerke R^2		0.041	

*p < .05; **p < .01; ***p < .001.

Table S6. Ordinal logistic regression models testing academic background and gender as determinants of how technologies should be regulated.

Ordinal logistic regression models						
	AGT			NT		
	Estimate	SE	Odds	Estimate	SE	Odds
Treshhold 1	-3.830***	.277		-2.961***	.184	
Treshhold 2	-.384***	.102		.533***	.104	
Treshhold 3	2.598***	.147		4.811***	.347	
Gender	.358**	.135	1.43	.114	.137	1.12
Humanities and social sciences	.770***	.188	2.16	.894***	.186	44
Nagelkerke R^2		0.033			0.034	
Parallel lines		Yes			Yes	

*p < .05; **p < .01; ***p < .001.

Note. Odds ratios presented above, as well as in subsequent ordinal regression tables, were computed as exp(b).

Table S6 cont. Ordinal logistic regression models testing academic background and gender as determinants of how technologies should be regulated.

Ordinal logistic regression models						
	SB			CT		
	Estimate	SE	Odds	Estimate	SE	Odds
Treshhold 1	-3.556***	.232		-1.675***	.122	
Treshhold 2	.150	.102		-.069	.100	
Treshhold 3	4.155***	.267		4.711***	.330	
Gender	.063	.136	1.07	.494***	.135	1.64
Humanities and social sciences	.746***	.188	2.11	-0.053	.186	0.95
Nagelkerke R^2		0.023			0.018	
Parallel lines		Yes			Yes	

*p < .05; **p < .01; ***p < .001.

Fig S1. Effect of academic background on how technologies should be regulated:

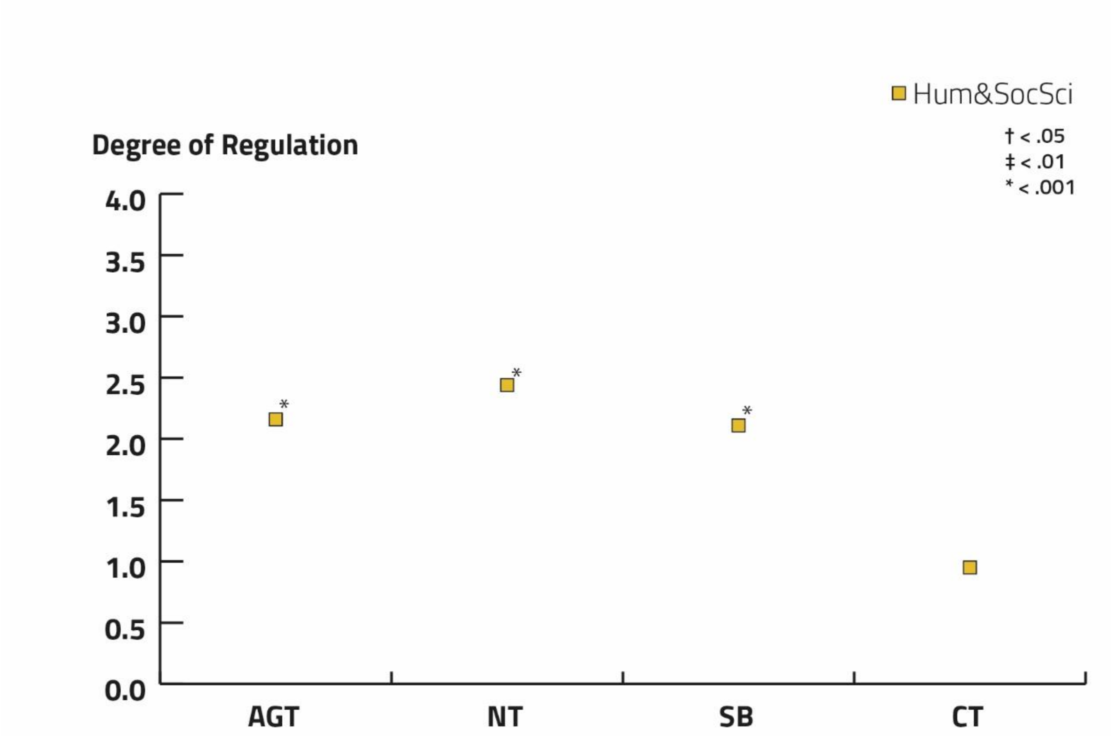

Fig. S1: Effects of academic background on how technologies should be regulated for NT, SB, AGT and CT using ordinal regression models. If models did not satisfy the test of parallel lines, a multinomial logistic regression was performed. As a reference category, natural sciences were selected. Significant results are indicated.

Fig S2. Effect of gender on how technologies should be regulated:

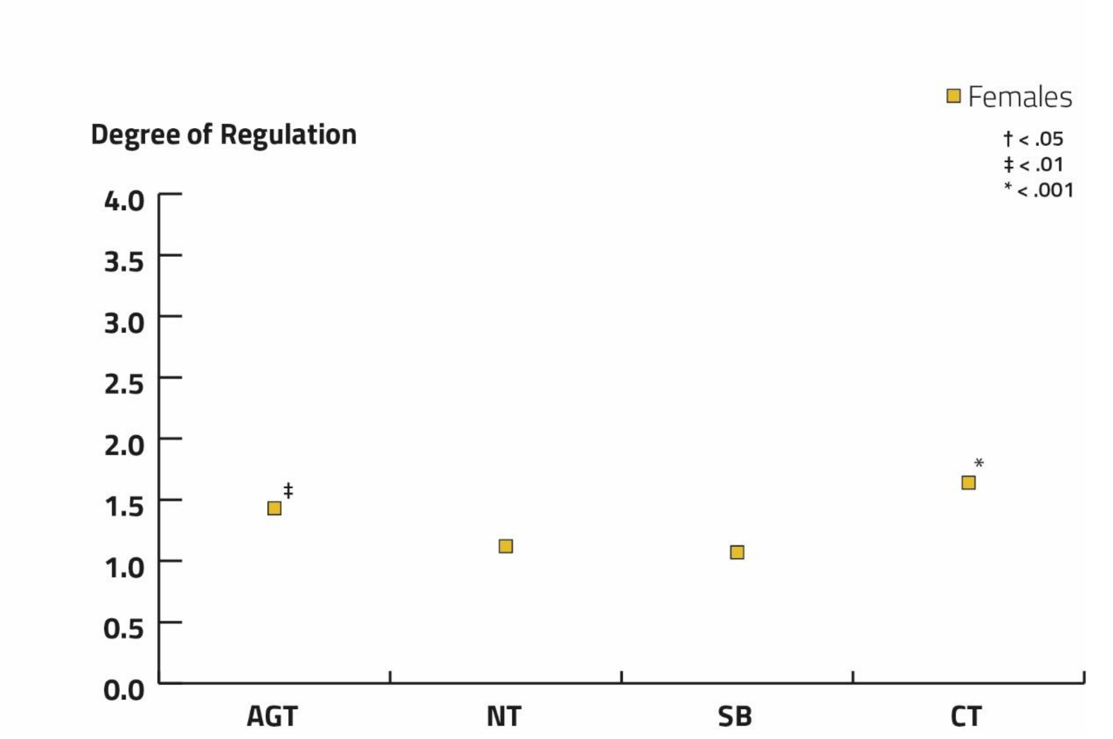

Fig. S2: Effects of gender on how technologies should be regulated for NT, SB, AGT and CT using ordinal regression models. If models did not satisfy the test of parallel lines, a multinomial logistic regression was performed. As a reference category, males were selected. Significant results are indicated.
